# Copper-Catalyzed Azide–Alkyne Cycloaddition-Oriented Multifunctional Bio-Orthogonal Linker BPPA: Design, Synthesis and Evaluation

**DOI:** 10.3390/molecules28248083

**Published:** 2023-12-14

**Authors:** Shuo Wang, Xu He, Junchen Li, Enxue Shi

**Affiliations:** State Key Laboratory of NBC Protection for Civilian, Beijing 102205, China; ws20140506@163.com (S.W.); hxalltags@163.com (X.H.)

**Keywords:** bio-orthogonal linker, phenacyl ester motif, click-cleavage evaluation, CuAAC reaction

## Abstract

The multifunctional linker molecules are crucial for the bio-orthogonal reaction for proteomic target profiling. Herein, we wish to present a novel type of biotin-based tetra-functional bio-orthogonal linkers **3a–3h** named BPPA which, possessing a unique photolabile phenacyl ester motif, were readily prepared in 85–90% yields by a simple and green one-step protocol from commercially available and inexpensive reagents of biotin acids and 4’-ethynyl/azido 2-bromoacetophenones. The typical click reaction of BPPA linkers **3a** and **3e** via copper-catalyzed azide–alkyne cycloaddition (CuAAC) took place easily, resulting in the corresponding BPPA-triazole adducts **4a** and **4b** in nearly quantitative yields. A further cleavability evaluation of **4a** and **4b** demonstrated that the expected C-O bond detachment could be accomplished efficiently and rapidly by UV irradiation or by ammonia hydrolysis, respectively, resulting in the residual (hydroxyl)acetylphenyl triazole fragment supposed to be attached to proteins during biological manipulations. The BPPA linkers, with dual clickable options of either the terminal azide or alkyne clickable group, exhibit high potentials for various CuAAC-oriented bio-orthogonal reactions.

## 1. Introduction

Dynamically investigating the life processes of biomolecules in the natural environment with chemical methods has always attracted significant attention. However, it is difficult to find chemical reactions with good biocompatibility and high selectivity under physiological conditions or in cells of living organisms due to the vast complexity of biological systems [1]. Fortunately, the bio-orthogonal reaction has offered an unprecedented opportunity for the study and manipulation of biological processes within living systems and attracted a surge of interest since its first appearance in 2003 [2,3]. An orthogonally modified biomolecule, which requires the presence of a reactive group as a handle for the attachment, could enable conjugation in a great variety of sequences, independent of each other, thereby serving as an ideal framework for heterobifunctional cross-linking to investigate their functionality in situ. In a typical proteomic workflow, affinity enrichment of the modified and conjugated sequences using the avidin–biotin interaction remains a commonly utilized strategy to isolate and enrich target proteins from the complex proteome [4]. The so-called bio-orthogonal linkers, generally accessible via chemical synthesis, are then widely employed in such chemical proteomic research. Of note, biotin-based bio-orthogonal linkers are in widespread use due to the fact that the water-soluble biotin can form an extraordinary stable complex with avidin, travidin or streptavidin (Avn-Btn), which is capable of performing efficient protein enrichment for proteomic target profiling [5,6]. However, the separation of biotinylated ligands or ligand–receptor complexes from the avidin matrix always requires harsh conditions, which may induce conformational changes and a loss of functions in the target molecules [7,8]. In order to circumvent the limitations of heat-induced avidin denaturation, multifunctional bio-orthogonal linkers, especially incorporating a readily cleavable motif to facilitate the release of target proteins, have emerged as a powerful tool for studying protein function and dynamics in recent decades [9,10,11]. In recent years, a large number of chemically cleavable linkers have also been developed. These existing click-chemistry-compatible cleavable linkers facilitate the enrichment of labeled proteins and peptides from complex mixtures with subsequent release under mild conditions for mass spectrometric analysis [12].

Among diverse bio-orthogonal reactions, the copper-catalyzed azide–alkyne cycloaddition (CuAAC) is the only ligation wherein both reactive tags, namely azide (–N_3_) and alkyne (–C≡CH), can be switched on the chemical reporter or on the linker, making this reaction very flexible and adaptable to various labeling strategies [13]. By comparison, only azide is adapted for SPAAC bio-orthogonal ligation without copper-associated cytotoxicity, but generally suffering from poorer kinetics and specificity, especially in the intracellular environment [14]. Therefore, azide or alkyne-featured linker molecules have been developed as the most popular choice so far. Particularly, the light-induced cleavage of chemically masked groups contained in linkers have drawn considerable interest for some time because such procedures assume that a photo-chemically cleavable biotin linker may afford clean and useful methodologies for the isolation of intact ligand-receptor complexes [15,16,17]. Despite the remarkable improvements and diverse applications that have been reported, the use of readily accessible and commercially available CuAAC-oriented bio-orthogonal linkers is still very limited so far, which could be partly ascribed to the persisting challenges connected with their synthesis and structural modification [18,19].

**Figure 1:**
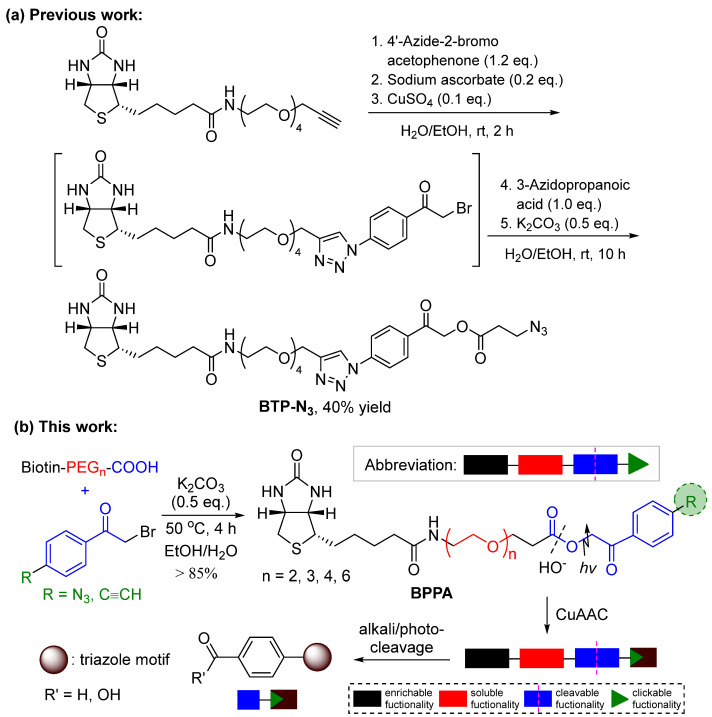
The CuAAC-oriented multifunctional bio-orthogonal linkers. (**a**) Previous work-design and synthesis of linker BTP-N_3_ [20]. The chemical synthesis starts from biotin-PEG_4_-alkyne to form the triazole intermediate which further reacts with 3-azidopropanoic acid to give the final product in 40% yield; (**b**) This work-design and synthesis of linker BPPA. BPPA was obtained by an one-step reaction between biotin-PEG_n_-COOH and 4′-azido(alkynyl)-2-bromoacetophenone in yields of 85–90%.

In our recent work, we developed an azide-type linker BTP-N_3_ for the most utilized CuAAC cycloaddition in this area and succeeded to achieve the click–enrich–release–identify workflow for the phosphonylated adducts’ identification with alkyne-tagged organophosphorus V-type warfare agents [20]. However, to obtain the phenacyl ester motif, a two-step reaction and some expensive reagents must be employed, whereas the BTP-N_3_ only results in a 40% overall yield (Figure 1a). After a careful re-consideration, we supposed that the key photo-cleavable phenacyl ester structure should be achieved when using a commercial biotin acid instead of the biotin alcohol to react directly with the 2-bromoacetophenone substrate comprising another clickable moiety in the molecule. Evidently, this would simplify the synthetic procedure and significantly benefit the economy efficiency. Herein, we aimed to report a new modified bio-orthogonal linker which could be prepared much more conveniently from cheap reagents (Figure 1b).

This novel CuAAC-oriented bio-orthogonal linker, herein named as BPPA, was assembled with four characteristic functionalities (expressed and abbreviated in different colors in Figure 1b): (a) the biotin scaffold for biologic enrichment, (b) the polyethylene glycol (PEG) chain for water dissolution, (c) the phenacyl ester motif for further cleavage and (d) the coupling azide or alkyne functional group for CuAAC reaction. To be emphasized, both the clickable azide (–N_3_) and the alkyne (–C≡CH) groups could be introduced onto the BPPA’s framework with identical efforts in our practical synthetic approach just by using a different 2-bromoacetophenone substrate as the starting material.

## 2. Results and Discussion

According to our previous experiments [20], the reaction between biotin-PEG_2_-COOH **1a** and 4’-azido-2-bromoacetophenone **2a** in the presence of potassium carbonate (0.5 eq.) was first examined. After stirring for 4 h at 50 °C in a green EtOH/H_2_O (1:1) solvent system, LC-MS monitoring showed that the starting materials were completely converted into the target compound BPPA **3a**. An ultrasound manipulation was generally needed to dissolve the biotin substrate. After removal of the solvent at the end of the reaction, the crude product was purified by flash column chromatography to give **3a** in 89% yield (Table 1, entry 1). Alternatively, the high pure product **3a** could also be obtained in a compatible yield by a column-free procedure simply via extraction with chloroform followed with washing by H_2_O. Hence, other BPPA molecules, namely **3b–3h**, were also prepared in compatible yields of 85–90% by this benign protocol with 4’-azido-2-bromoacetophenone **2a** or 4’-ethynyl-2-bromoacetophenone **2b**, as shown in Table 1 (entries 2–8). Of note, the spacer length of the PEG chain, which determines the water solubility of the linkers, exhibited almost no influence on the synthetic efficiency for BPPA and could be easily adjusted only by changing the starting biotin-PEG_n_-COOH to complete different proteomic experiments [21].

**Scheme 1:**
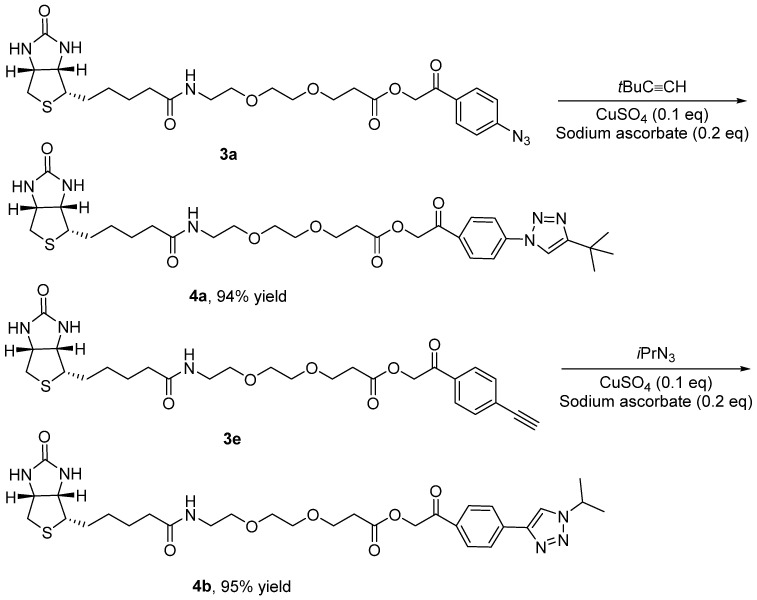
The CuAAC reaction of BPPA **3a** and **3e**.

With the BPPA linkers in hand, we then investigated their applicability for bio-orthogonal ligation and the cleavage efficiency under a representative chemical circumstance (Scheme 1). The azide linker **3a** as well as the alkyne linker **3e** were both tested. Under the typical CuAAC conditions [22] by using catalytic CuSO_4_ (0.1 eq.) and sodium ascorbate (0.2 eq.), compound **3a** and **3e** were transformed into the BPPA-triazole heterocyclic adducts **4a** and **4b,** respectively, both in nearly quantitative isolated yields, as shown in Scheme 1. It is noteworthy that one of the most steric *tert*-butyl acetylene has been examined in the CuAAC reaction with excellent reactivity, which strongly indicates that other bulky substituents should also be applicable in typical CuAAC reaction conditions.

**Figure 2:**
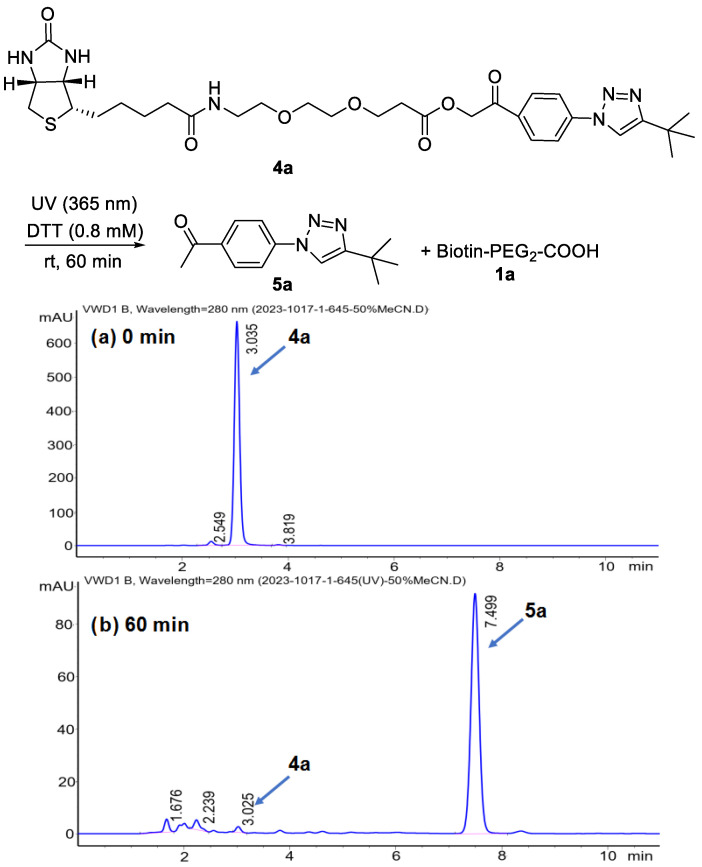
HPLC analysis of the photo-promoted cleavage reaction of BPPA-triazole **4a**. (**a**) the starting material; (**b**) irradiation after 60 min.

To explore the potential of the BPPA linkers, we firstly evaluated the more favorable photo-promoted hydrolysis cleavage reaction of BPPA **4a** (Figure 2). For the irradiation experiments, 0.8 mM dithilthreitol (DTT) was added as a photo scavenger to neutralize the free radicals generally formed during the UV irradiation [23]. It was found that, simply by placing a common 12 W UV lamp upon the sample in the solvent of CH_3_CN/H_2_O (1:1) and irradiating BPPA **4a** for 60 min at 365 nm, nearly 100% of the expected product **5a** from the cleavage of the C(O)O–C bond of the phenacyl ester motif was determined by HPLC analysis (Figure 2b). It is worth noting that the additive of DTT was necessary for the photo procedure; otherwise, the linker **4a** would be oxidized into the corresponding sulfoxide form during the UV irradiation, which then would lead to an absence of the expected cleavage reaction.

**Figure 3:**
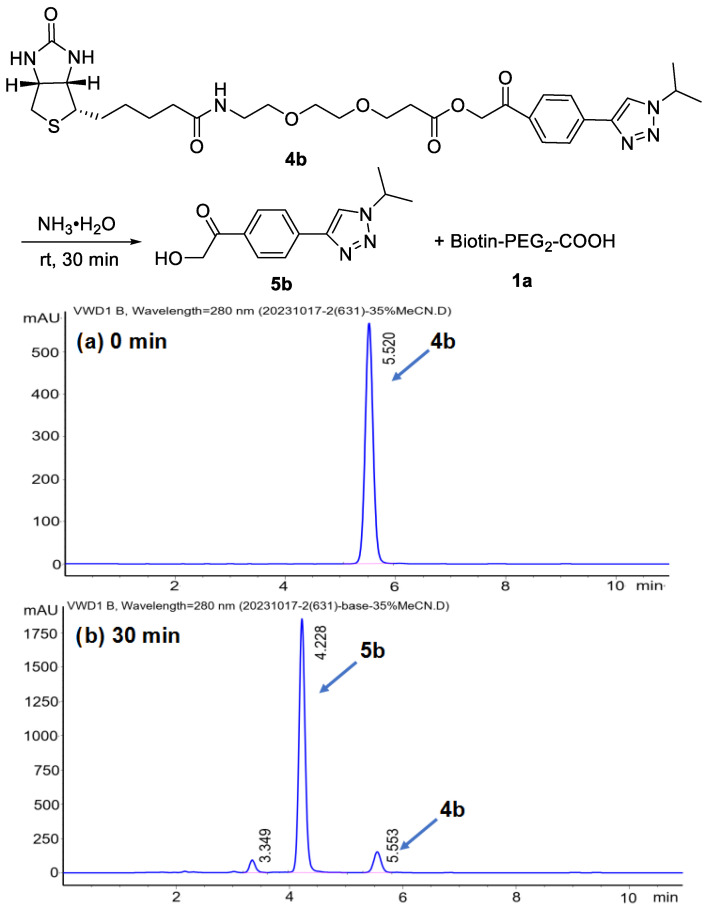
HPLC analysis of the alkali-promoted cleavage reaction of BPPA-triazole **4b**. (**a**) the starting material; (**b**) hydrolysis after 30 min.

Next, the chemical dissociation feasibility of the BPPA-derived triazole molecule **4b** was also examined, as shown in Figure 3. It was found that, after stirring for only 30 min at room temperature with aqueous ammonia (15%), 85% of compound **4b** had been converted into α-hydroxyl acetophenone product **5b** via the detachment of the C(O)–OC bond in the phenacyl ester motif, according to the HPLC analysis (Figure 3b). Interestingly, the phenyltriazole products **5a** and **5b** generated from the above two cleavage pathways, though featuring with similar structures, displayed very different HPLC retention times (RT) of 7.499 min versus 4.228 min, respectively, which is probably due to the polar hydroxyl group introduced in **5b**.

**Figure 4:**
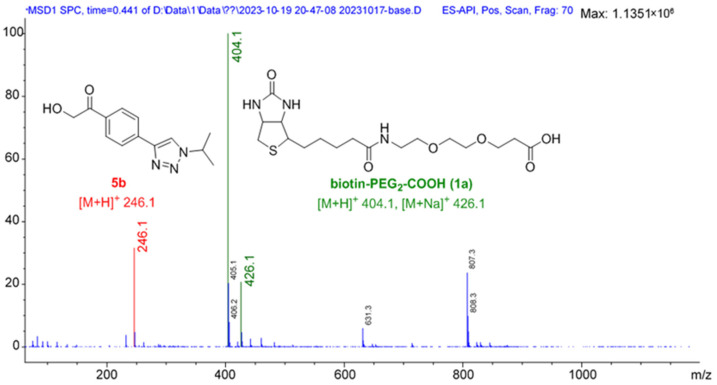
LC-MS analysis of the ammonia cleavage products of BPPA-triazole **4b**.

The biotin-containing product formed in the two cleavage reactions was not detected by HPLC since it has no UV absorption. Hence, the presumed biotin-PEG_2_-COOH was confirmed by a further LC-MS analysis, which suggests that the starting biotin acids could be recovered in the release manipulations. Of note, only biotin-PEG_2_-COOH rather than biotin-PEG_2_-CONH_2_ was observed in the resulted mixture from ammonia hydrolysis (Figure 4). The HPLC and LC-MS analysis results demonstrate that both cleavage pathways proceeded rapidly and cleanly and that almost no side-products were generated, except for the recovered biotin acid material.

Compared to the base-involved cleavage procedure, the photo-mediated approach appeared to be advantageous due to its easy handling similar reaction kinetics were found. It is clear that, in the two cleavage ways seen above, the final residual fragments of (hydroxyl)acetylphenyl triazole, which are supposed to be left behind on a target protein, are very close in molecular weights.

**Scheme 2:**
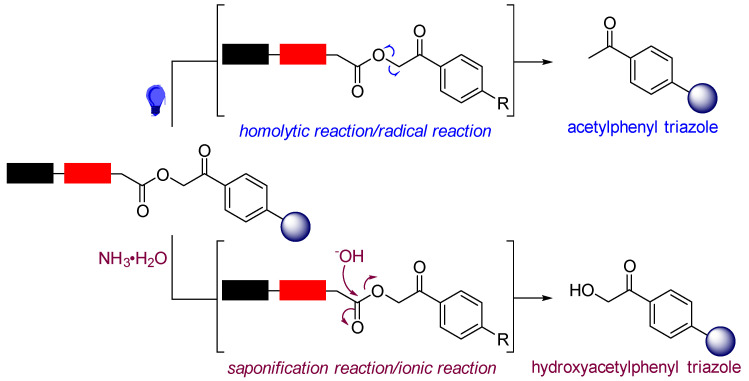
Proposed mechanism for the photo/alkali-cleavage reactions of BPPA-triazole. The black rectangular indicates the enrichable functionality, whereas the red rectangular indicates the soluble functionality, which has also been described in Figure 1.

A plausive mechanism for the photo/alkali-cleavage reactions of BPPA-triazole was given in Scheme 2. Based on previous reports about the photo-mediated selective cleavage of the phenacyl ester [15], the photo-cleavage of the C_sp3_-O bond in BPPA-triazole should proceed via a homolytic radical reaction to give acetylphenyl triazole **5a**. Meanwhile, in the presence of an alkaline ammonia solution, a classical ionic saponification of the C(O)-O bond in the ester group of BPPA-triazole should occur to give hydroxyacetylphenyl triazole **5b**.

## 3. Experimental

All the commercially available reagents were used without further purification unless otherwise stated. Photo-cleavage experiments were conducted with a Steema 12W UV lamp. LC-MS monitoring was conducted on an Agilent LC-MSD instrument (Santa Clara, CA, USA). HPLC analysis was conducted on an Agilent 1260 LC/MSC with a MZ PerfectChrom 100 C8 column (4.6 ID × 150 mm, 5 µm). ^1^H and ^13^C NMR spectra were recorded on a Bruker 300 instrument and were calibrated using residual undeuterated solvent (Chloroform-*d* @ 7.26 ppm, ^1^H NMR; Chloroform-*d* @ 77.16 ppm, ^13^C NMR). All ^1^H NMR spectra were reported in delta (δ) units, parts per million (ppm) downfield from the internal standard. Coupling constants were reported in Hertz (Hz). High-resolution mass spectra (HRMS) were obtained from an Agilent 6545 Q-TOF HPLC-MS mass spectrometer (Santa Clara, CA, USA) by electrospray ionization time of flight reflectron experiments. FTIR spectra were recorded with a Bruker VERTEX70 Tango-R spectrophotometer (Billerica, MA, USA). UV/Vis spectra were recorded with a Shimadzu UV-2550 spectrophotometer (Kyoto, Japan).

(1) General procedure for synthesis of BPPA linker **3**

To a solution of biotin-PEG_n_-COOH **1** (0.2 mmol) and K_2_CO_3_ (0.1 mmol) in H_2_O (1 mL) was added a solution of 4-ethynylbenzoyl bromide (0.2 mmol) or 4-azidobenzoyl bromide (0.2 mmol) **2** in EtOH (1 mL) under air atmosphere. After stirring at 50 °C for 4 h, column chromatography separation (CH_2_Cl_2_: CH_3_OH = 10:1) gave the target products **3**.

Data for compound **3a**: Faint yellow solid, yield 89%, 250 mg; ^1^H NMR (300 MHz, Chloroform-*d*) δ 7.92 (d, *J* = 8.7 Hz, 2H), 7.11 (d, *J* = 8.7 Hz, 2H), 6.87 (t, *J* = 5.3 Hz, 1H), 6.65 (s, 1H), 5.63 (s, 1H), 5.35 (s, 2H), 4.52–4.44 (m, 1H), 4.33–4.25 (m, 1H), 3.83 (t, *J* = 6.2 Hz, 2H), 3.66–3.52 (m, 6H), 3.50–3.35 (m, 2H), 3.10 (q, *J* = 7.2 Hz, 1H), 2.94–2.67 (m, 4H), 2.20 (t, *J* = 7.5 Hz, 2H), 2.08 (s, 1H), 1.79–1.52 (m, 4H), 1.50–1.32 (m, 2H). ^13^C NMR (75 MHz, Chloroform-*d*) δ 190.52, 173.33, 171.08, 163.99, 145.81, 130.54, 129.73, 119.30, 77.42, 77.00, 76.58, 70.07, 69.95, 66.25, 65.97, 61.67, 60.14, 55.54, 40.52, 39.10, 35.90, 34.69, 28.11, 28.03, 25.56. HRMS (*m*/*z*) calcd for C_25_H_34_N_6_O_7_S [M + H]^+^ 563.2282, found 563.2282. FTIR (cm^−1^) 3283.99, 3112.11, 2927.82, 2874.74, 2411.55, 2256.56, 2117.30, 1742.04, 1702.81, 1644.15, 1597.15, 1551.31, 1503.79, 1462.71, 1423.04, 1370.98, 1283.41, 1239.94, 1173.71, 1118.17, 972.02, 889.83, 831.60, 759.39, 724.01, 693.63,651.74. UV/Vis (nm) λ_max_ = 289.50.

Data for compound **3b**: Faint yellow solid, yield 90%, 272 mg; ^1^H NMR (300 MHz, Chloroform-*d*) δ 7.89 (d, *J* = 8.7 Hz, 2H), 7.09 (d, *J* = 8.7 Hz, 2H), 6.98 (t, *J* = 5.2 Hz, 1H), 6.77 (s, 1H), 5.90 (s, 1H), 5.31 (s, 2H), 4.51–4.42 (m, 1H), 4.32–4.22 (m, 1H), 3.79 (t, *J* = 6.4 Hz, 2H), 3.61 (d, *J* = 4.9 Hz, 8H), 3.53 (t, *J* = 4.9 Hz, 2H), 3.43–3.37 (m, 2H), 3.09 (q, *J* = 7.1 Hz, 1H), 2.91–2.64 (m, 5H), 2.18 (t, *J* = 7.4 Hz, 2H), 1.64 (tt, *J* = 13.6, 7.1 Hz, 4H), 1.39 (q, *J* = 7.3 Hz, 2H). ^13^C NMR (75 MHz, Chloroform-*d*) δ 190.48, 173.41, 170.95, 164.17, 145.68, 130.57, 129.66, 119.20, 77.42, 77.00, 76.58, 70.27, 70.22, 69.95, 69.87, 66.30, 65.85, 61.64, 60.13, 55.61, 40.44, 39.03, 35.86, 34.60, 28.17, 27.98, 25.56. HRMS (*m*/*z*) calcd for C_27_H_38_N_6_O_8_S [M + H]^+^ 607.2545, found 607.2556. FTIR (cm^−1^) 3332.63, 3238.58, 2936.25, 2411.19, 2247.68, 2113.27, 1749.36, 1698.87, 1659.87, 1598.41, 1573.27, 1506.85, 1454.52, 1422.10, 1373.90, 1331.29, 1287.02, 1236.94, 1175.64, 1096.09, 967.27, 906.04, 836.40, 724.20, 646.88. UV/Vis (nm) λ_max_ = 290.00.

Data for compound **3c**: Faint yellow solid, yield 87%, 282 mg; ^1^H NMR (300 MHz, Chloroform-*d*) δ 7.88 (d, *J* = 8.5 Hz, 2H), 7.07 (d, *J* = 8.5 Hz, 2H), 7.01 (t, *J* = 5.1 Hz, 1H), 6.80 (s, 1H), 5.98 (s, 1H), 5.29 (s, 2H), 4.51–4.40 (m, 1H), 4.31–4.20 (m, 1H), 3.78 (t, *J* = 6.5 Hz, 2H), 3.67–3.46 (m, 14H), 3.42–3.31 (m, 2H), 3.08 (q, *J* = 6.8 Hz, 1H), 2.94–2.64 (m, 5H), 2.18 (t, *J* = 7.3 Hz, 2H), 1.75–1.52 (m, 4H), 1.38 (q, *J* = 7.0 Hz, 2H). ^13^C NMR (75 MHz, Chloroform-*d*) δ 190.45, 173.35, 170.85, 164.17, 145.60, 130.50, 129.62, 119.14, 77.42, 77.00, 76.58, 70.36, 70.26, 70.19, 69.88, 69.84, 66.26, 65.79, 61.59, 60.10, 55.60, 40.39, 38.97, 35.81, 34.54, 28.16, 27.96, 25.53. HRMS (*m*/*z*) calcd for C_29_H_42_N_6_O_9_S [M + H]^+^ 651.2807, found 651.2807. FTIR (cm^−1^) 3286.19, 2928.40, 2413.29, 2248.21, 2113.04, 1745.78, 1699.66, 1598.90, 1507.06, 1459.67, 1423.09, 1372.84, 1286.47, 1237.70, 1175.12, 1096.00, 970.12, 905.48, 829.16, 723.83, 647.30. UV/Vis (nm) λ_max_ = 289.40.

Data for compound **3d**: Faint yellow solid, yield 85%, 313 mg; ^1^H NMR (300 MHz, Chloroform-*d*) δ 7.86 (d, *J* = 7.9 Hz, 2H), 7.05 (d, *J* = 7.9 Hz, 2H), 6.95 (s, 1H), 6.84 (s, 1H), 6.06 (s, 1H), 5.26 (s, 2H), 4.42 (s, 1H), 4.23 (s, 1H), 3.75 (t, *J* = 5.7 Hz, 2H), 3.57 (s, 24H), 3.35 (s, 2H), 3.12–2.97 (m, 1H), 2.74 (td, *J* = 24.0, 21.8, 12.3 Hz, 4H), 2.15 (s, 2H), 1.76–1.48 (m, 4H), 1.42–1.30 (m, 2H). ^13^C NMR (75 MHz, Chloroform-*d*) δ 190.37, 173.23, 170.77, 164.14, 145.48, 130.44, 129.54, 119.04, 77.43, 77.00, 76.58, 70.28, 70.19, 70.16, 70.09, 69.80, 69.73, 66.17, 65.69, 61.51, 60.03, 55.56, 40.30, 38.89, 35.75, 34.48, 28.12, 27.89, 25.44. HRMS (*m*/*z*) calcd for C_33_H_50_N_6_O_11_S [M + H]^+^ 739.3331, found 739.3340. FTIR (cm^−1^) 3300.51, 2873.50, 2246.75, 1746.49, 1701.71, 1657.26, 1604.83, 1523.20, 1459.45, 1425.07, 1372.55, 1329.88, 1228.78, 1173.03, 1096.17, 970.90, 905.54, 826.06, 723.85, 647.32. UV/Vis (nm) λ_max_ = 270.50.

Data for compound **3e**: Faint yellow solid, yield 90%, 245 mg; ^1^H NMR (300 MHz, Chloroform-*d*) δ 7.87 (d, *J* = 8.4 Hz, 2H), 7.59 (d, *J* = 8.3 Hz, 2H), 6.84 (t, *J* = 5.2 Hz, 1H), 6.62 (s, 1H), 5.63 (s, 1H), 5.36 (s, 2H), 4.54–4.43 (m, 1H), 4.34–4.23 (m, 1H), 3.82 (t, *J* = 6.2 Hz, 2H), 3.68–3.50 (m, 6H), 3.50–3.35 (m, 2H), 3.30 (s, 1H), 3.10 (q, *J* = 7.2 Hz, 1H), 2.93–2.66 (m, 4H), 2.20 (t, *J* = 7.4 Hz, 2H), 2.07 (s, 1H), 1.77–1.56 (m, 4H), 1.46–1.32 (m, 2H). ^13^C NMR (75 MHz, Chloroform-*d*) δ 191.33, 173.33, 171.04, 163.98, 133.61, 132.54, 127.65, 82.43, 81.11, 77.42, 77.00, 76.58, 70.09, 69.94, 66.24, 66.13, 61.68, 60.14, 55.55, 40.52, 39.10, 35.90, 34.68, 28.13, 28.03, 25.57. HRMS (*m*/*z*) calcd for C_27_H_35_N_3_O_7_S [M + H]^+^ 546.2268, found 546.2268. FTIR (cm^−1^) 3285.35, 3083.49, 2874.44, 1741.05, 1699.75, 1644.27, 1603.63, 1551.93, 1462.06, 1424.24, 1404.59, 1370.77, 1320.89, 1281.38, 1232.26, 1187.69, 1172.87, 1123.47, 1022.69, 1008.83, 972.63, 857.06, 827.16, 761.56, 726.30, 700.43, 652.04. UV/Vis (nm) λ_max_ = 271.00.

Data for compound **3f**: Faint yellow solid, yield 87%, 256 mg; ^1^H NMR (300 MHz, Chloroform-*d*) δ 7.88 (d, *J* = 8.4 Hz, 2H), 7.59 (d, *J* = 8.4 Hz, 2H), 7.01 (t, *J* = 5.3 Hz, 1H), 6.88 (s, 1H), 6.07 (s, 1H), 5.36 (s, 2H), 4.55–4.44 (m, 1H), 4.35–4.24 (m, 1H), 3.82 (t, *J* = 6.4 Hz, 2H), 3.71–3.51 (m, 10H), 3.50–3.37 (m, 2H), 3.34 (s, 1H), 3.12 (q, *J* = 7.1 Hz, 1H), 2.96–2.68 (m, 5H), 2.22 (t, *J* = 7.4 Hz, 2H), 1.81–1.56 (m, 4H), 1.42 (q, *J* = 7.2 Hz, 2H). ^13^C NMR (75 MHz, Chloroform-*d*) δ 191.40, 173.50, 170.99, 164.36, 133.68, 132.52, 127.79, 127.69, 82.48, 81.23, 77.57, 77.14, 76.72, 70.36, 70.34, 70.31, 70.04, 69.96, 66.38, 66.11, 61.74, 60.24, 55.75, 40.53, 39.12, 35.98, 34.69, 28.32, 28.09, 25.67. HRMS (*m*/*z*) calcd for C_29_H_39_N_3_O_8_S [M + H]^+^ 590.2531, found 590.2545. FTIR (cm^−1^) 3299.52, 2926.41, 2871.10, 2246.11, 2106.86, 1745.84, 1699.96, 1656.58, 1604.47, 1528.94, 1459.00, 1424.91, 1372.99, 1330.60, 1227.73, 1172.84, 1098.79, 970.85, 906.15, 825.55, 724.64, 646.99. UV/Vis (nm) λ_max_ = 270.70.

Data for compound **3g**: Faint yellow solid, yield 88%, 279 mg; ^1^H NMR (300 MHz, Chloroform-*d*) δ 7.83 (d, *J* = 8.2 Hz, 2H), 7.54 (d, *J* = 8.2 Hz, 2H), 6.96 (t, *J* = 4.9 Hz, 1H), 6.83 (s, 1H), 6.01 (s, 1H), 5.30 (s, 2H), 4.50–4.40 (m, 1H), 4.31–4.19 (m, 1H), 3.77 (t, *J* = 6.4 Hz, 2H), 3.66–3.48 (m, 14H), 3.41–3.26 (m, 3H), 3.07 (q, *J* = 6.7 Hz, 1H), 2.94–2.65 (m, 4H), 2.17 (t, *J* = 7.3 Hz, 2H), 1.75–1.53 (m, 4H), 1.38 (q, *J* = 7.0 Hz, 2H). ^13^C NMR (75 MHz, Chloroform-*d*) δ 191.26, 173.31, 170.81, 164.19, 133.56, 132.37, 127.63, 127.55, 82.34, 81.06, 77.42, 77.00, 76.58, 70.37, 70.25, 70.17, 69.89, 69.82, 66.24, 65.95, 61.58, 60.10, 55.61, 40.38, 38.97, 35.84, 34.54, 28.17, 27.96, 25.52. HRMS (*m*/*z*) calcd for C_31_H_43_N_3_O_9_S [M + H]^+^ 634.2793, found 634.280. FTIR (cm^−1^) 3300.03, 2926.79, 2872.81, 2246.13, 1746.16, 1700.68, 1656.78, 1604.69, 1525.38, 1459.17, 1425.09, 1372.65, 1330.39, 1228.08, 1172.99, 1095.66, 971.03, 905.99, 825.93, 724.20, 646.97. UV/Vis (nm) λ_max_ = 271.60.

Data for compound **3h**: Faint yellow solid, yield 86%, 310 mg; ^1^H NMR (300 MHz, Chloroform-*d*) δ 7.79 (d, *J* = 8.3 Hz, 2H), 7.50 (d, *J* = 8.2 Hz, 2H), 6.95 (t, *J* = 5.1 Hz, 1H), 6.84 (s, 1H), 6.09 (s, 1H), 5.26 (s, 2H), 4.45–4.36 (m, 1H), 4.26–4.16 (m, 1H), 3.74 (t, *J* = 6.5 Hz, 2H), 3.64–3.44 (m, 23H), 3.38–3.27 (m, 3H), 3.10–2.98 (m, 1H), 2.84–2.60 (m, 4H), 2.13 (t, *J* = 7.3 Hz, 2H), 1.61 (dd, *J* = 13.5, 6.5 Hz, 4H), 1.33 (d, *J* = 14.4 Hz, 2H). ^13^C NMR (75 MHz, Chloroform-*d*) δ 191.15, 173.20, 170.69, 164.13, 133.44, 132.22, 127.48, 82.22, 81.07, 77.43, 77.00, 76.57, 70.24, 70.15, 70.12, 70.04, 69.75, 69.68, 66.11, 65.82, 61.47, 59.98, 55.54, 40.25, 38.85, 35.71, 34.43, 28.10, 27.85, 25.41. HRMS (*m*/*z*) calcd for C_35_H_51_N_3_O_11_S [M + H]^+^ 722.3317, found 722.3323. FTIR (cm^−1^) 3315.26, 2873.15, 2411.57, 2246.31, 2113.06, 1745.86, 1699.60, 1657.91, 1598.83, 1506.99, 1458.68, 1423.11, 1372.77, 1286.46, 1238.30, 1175.08, 1095.55, 969.75, 906.03, 830.18, 724.08, 646.94. UV/Vis (nm) λ_max_ = 290.00.

(2) General procedure for synthesis of BPPA-triazole **4**

To a solution of **3a** (0.2 mmol) and 3,3-dimethylbut-1-yne (0.2 mmol) in EtOH (1 mL) was added a solution of natrascorb (0.04 mmol) and CuSO_4_ (0.02 mmol) in H_2_O (1 mL). After stirring at rt for 1 h, **4a** was obtained as a white solid after HPLC purification using a C8 (30 × 250 mm) column with CH_3_CN/H_2_O (35/65) as a mobile phase. For compound **4b**, **3e** (0.2 mmol) and 2-azidopropane (0.2 mmol) were used.

Data for compound **4a**: White solid, yield 94%, 121 mg; ^1^H NMR (300 MHz, Chloroform-*d*) δ 8.07 (d, *J* = 8.6 Hz, 2H), 7.91 (d, *J* = 8.6 Hz, 2H), 7.83 (s, 1H), 6.95 (t, *J* = 5.1 Hz, 1H), 6.70 (s, 1H), 5.70 (s, 1H), 5.41 (s, 2H), 4.51–4.43 (m, 1H), 4.31–4.23 (m, 1H), 3.83 (t, *J* = 6.1 Hz, 2H), 3.68–3.51 (m, 6H), 3.46–3.36 (m, 2H), 3.09 (q, *J* = 7.0 Hz, 1H), 2.91–2.65 (m, 4H), 2.31–2.17 (m, 3H), 1.60 (dt, *J* = 13.8, 6.8 Hz, 4H), 1.41 (s, 11H). ^13^C NMR (75 MHz, Chloroform-*d*) δ 190.88, 173.40, 171.09, 164.07, 158.89, 140.90, 133.23, 129.57, 119.98, 116.57, 77.42, 77.00, 76.58, 70.10, 69.92, 66.22, 66.12, 61.66, 60.13, 55.55, 40.50, 39.09, 35.87, 34.66, 30.87, 30.17, 28.12, 28.02, 25.59. HRMS (*m*/*z*) calcd for C_31_H_44_N_6_O_7_S [M + H]^+^ 645.3065, found 645.3074. FTIR (cm^−1^) 3434.62, 3006.52, 2945.05, 2360.47, 2295.07, 2255.96, 2068.89, 1638.81, 1442.34, 1375.47, 1038.52, 920.69, 751.89, 685.42. UV/Vis (nm) λ_max_ = 281.95.

Data for compound **4b**: White solid, yield 95%, 119 mg; ^1^H NMR (300 MHz, Chloroform-*d*) δ 7.95 (s, 5H), 6.95 (t, *J* = 5.2 Hz, 1H), 6.68 (s, 1H), 5.77 (s, 1H), 5.39 (s, 2H), 4.88 (p, *J* = 6.7 Hz, 1H), 4.51–4.40 (m, 1H), 4.30–4.21 (m, 1H), 3.82 (t, *J* = 6.2 Hz, 2H), 3.66–3.50 (m, 6H), 3.46–3.34 (m, 2H), 3.07 (q, *J* = 7.1 Hz, 1H), 2.92–2.65 (m, 4H), 2.40 (s, 1H), 2.18 (t, *J* = 7.3 Hz, 2H), 1.63 (d, *J* = 6.7 Hz, 10H), 1.35 (dt, *J* = 13.4, 6.6 Hz, 2H). ^13^C NMR (75 MHz, Chloroform-*d*) δ 191.42, 173.41, 171.11, 164.08, 145.97, 136.17, 133.08, 128.43, 125.74, 118.47, 77.42, 77.00, 76.58, 70.06, 69.94, 69.91, 66.22, 66.16, 61.64, 60.12, 55.56, 53.25, 40.48, 39.07, 35.86, 34.67, 28.13, 28.01, 25.58, 23.01. HRMS (*m*/*z*) calcd for C_30_H_42_N_6_O_7_S [M + H]^+^ 631.2908, found 631.2912. FTIR (cm^−1^) 3289.01, 3103.11, 2930.32, 2869.16, 1745.06, 1697.97, 1611.52, 1543.06, 1456.70, 1425.43, 1372.87, 1332.14, 1225.46, 1177.36, 1115.83, 1038.09, 972.12, 826.66, 734.36. UV/Vis (nm) λ_max_ = 291.50.

## 4. Conclusions

By using bio-orthogonal chemistry as a key toolbox, versatile biotinylated linkers endowed with various cleavable functional groups were designed to selectively elute proteins of interest, the most impressive of which are those related to CuAAC reactions. Herein, we developed a new kind of biotin-based bio-orthogonal linkers named BPPA, which could be easily synthesized in high yields from commercially available reagents by a simple one-step reaction under environmental benign conditions. These multifunctional linking molecules, featuring the unique phenacyl ester motif, were proved to be highly efficient in the typical CuAAC ligation, as well as the photo/alkali-promoted selective C–O bond cleavage reactions. To be emphasized, the starting biotin acids could be recovered completely during the cleavage process.

More importantly, the BPPA linker represents a valuable complement to the bio-orthogonal toolkit with excellent flexibility in the choice of a reactive terminal azide or alkyne clickable group, which would significantly benefit the related proteomic experiments. We expect that the BPPA linker with dual CuAAC-ligation options will find applications in the following chemical biology studies.

## Figures and Tables

**Table 1:** One-step synthesis of bio-orthogonal linker BPPA **3a–3h** ^a^.

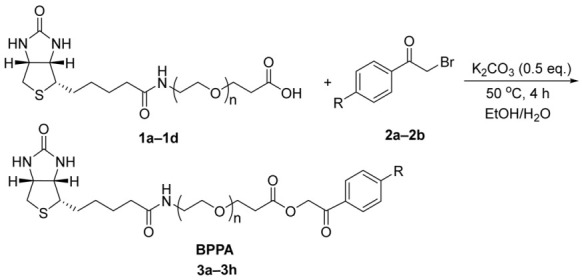				
Entry	BPPA	R	n	3 (%) ^b^
1	**3a**	–N_3_	2	89
2	**3b**	–N_3_	3	90
3	**3c**	–N_3_	4	87
4	**3d**	–N_3_	6	85
5	**3e**	–C≡CH	2	90
6	**3f**	–C≡CH	3	87
7	**3g**	–C≡CH	4	88
8	**3h**	–C≡CH	6	86

^a^ Reaction conditions: **1** (0.2 mmol), **2** (0.2 mmol), K_2_CO_3_ (0.1 mmol), EtOH (1 mL), H_2_O (1 mL), 50 °C, 4 h, in the air; ^b^ isolated yields.

## Data Availability

Data are contained within the article and Supplementary Materials.

## Supplementary Materials

The following supporting information can be downloaded at: https://www.mdpi.com/article/10.3390/molecules28248083/s1, Figure S1: ^1^H NMR of compound **3a**; Figure S2: ^13^C NMR of compound **3a**; Figure S3: ^1^H NMR of compound **3b**; Figure S4: ^13^C NMR of compound **3b**; Figure S5: ^1^H NMR of compound **3c**; Figure S6: ^13^C NMR of compound **3c**; Figure S7: ^1^H NMR of compound **3d**; Figure S8: ^13^C NMR of compound **3d**; Figure S9: ^1^H NMR of compound **3e**; Figure S10: ^13^C NMR of compound **3e**; Figure S11: ^1^H NMR of compound **3f**; Figure S12: ^13^C NMR of compound **3f**; Figure S13: ^1^H NMR of compound **3g**; Figure S14: ^13^C NMR of compound **3g**; Figure S15: ^1^H NMR of compound **3h**; Figure S16: ^13^C NMR of compound **3h**; Figure S17: ^1^H NMR of compound **4a**; Figure S18: ^13^C NMR of compound **4a**; Figure S19: ^1^H NMR of compound **4b**; Figure S20: ^13^C NMR of compound **4b**; Figure S21: The FTIR spectra of compound **3a**; Figure S22: The FTIR spectra of compound **3b**; Figure S23: The FTIR spectra of compound **3c**; Figure S24: The FTIR spectra of compound **3d**; Figure S25: The FTIR spectra of compound **3e**; Figure S26: The FTIR spectra of compound **3f**; Figure S27: The FTIR spectra of compound **3g**; Figure S28: The FTIR spectra of compound **3h**; Figure S29: The FTIR spectra of compound **4a**; Figure S30: The FTIR spectra of compound **4b**; Figure S31: The UV/Vis spectra of compound **3a**; Figure S32: The UV/Vis spectra of compound **3b**; Figure S33: The UV/Vis spectra of compound **3c**; Figure S34: The UV/Vis spectra of compound **3d**; Figure S35: The UV/Vis spectra of compound **3e**; Figure S36: The UV/Vis spectra of compound **3f**; Figure S37: The UV/Vis spectra of compound **3g**; Figure S38: The UV/Vis spectra of compound **3h**; Figure S39: The UV/Vis spectra of compound **4a**; Figure S40: The UV/Vis spectra of compound **4b**.

## References

[1] Schauenburg D., Weil T. (2023). Chemical Reactions in Living Systems. Adv. Sci..

[2] Hang H.C., Yu C., Kato D.L., Bertozzi C.R. (2003). A metabolic labeling approach toward proteomic analysis of mucin-type O-linked glycosylation. Proc. Natl. Acad. Sci. USA.

[3] Bertozzi C.R. (2023). A special virtual issue celebrating the 2022 Nobel Prize in chemistry for the development of click chemistry and bioorthogonal chemistry. ACS Cent. Sci..

[4] Pan S., Zhang H., Wang C., Yao S.C.L., Yao S.Q. (2016). Target identification of natural products and bioactive compounds using affinity-based probes. Nat. Prod. Rep..

[5] González M., Bagatolli L.A., Echabe I., Arrondo J.L.R., Argarana C.E., Cantor C.R., Fidelio G.D. (1997). Interaction of biotin with streptavidin: Thermostability and conformational changes upon binding. J. Biol. Chem..

[6] Freitag S., Le Trong I., Chilkoti A., Klumb L.A., Stayton P.S., Stenkamp R.E. (1998). Structural studies of binding site tryptophan mutants in the high-affinity streptavidin-biotin complex. J. Mol. Biol..

[7] Green N.M., Anfinsen C.B., Edsall J.T., Richards F.M. (1975). Avidin. Advances in Protein Chemistry.

[8] Finn F.M., Titus G., Montibeller J.A., Hofmann K. (1980). Hormone-receptor studies with avidin and biotinylinsulin-avidin complexes. J. Biol. Chem..

[9] Haugland R.P. (2002). Handbook of Fluorescent Probes and Research Products.

[10] Breitinger H.G.A., Gee K.R., Carpenter B.K., Hess G.P. (2003). Toward the development of new photolabile protecting groups that can rapidly release bioactive compounds upon photolysis with visible light. J. Org. Chem..

[11] Leriche G., Chisholm L., Wagner A. (2012). Cleavable linkers in chemical biology. Bioorg. Med. Chem..

[12] Beard H.A., Korovesis D., Chen S., Verhelst S.H. (2021). Cleavable linkers and their application in MS-based target identification. Mol. Omics..

[13] Li L., Zhang Z. (2016). Development and applications of the copper-catalyzed azide-alkyne cycloaddition (CuAAC) as a bioorthogonal reaction. Molecules.

[14] Rigolot V., Biot C., Lion C. (2021). To view your biomolecule, click inside the cell. Angew. Chem. Int. Ed..

[15] Orth R., Sieber S.A. (2009). A photolabile linker for the mild and selective cleavage of enriched biomolecules from solid support. J. Org. Chem..

[16] Yang Y., Verhelst S.H.L. (2013). Cleavable trifunctional biotin reagents for protein labelling, capture and release. Chem. Commun..

[17] Karaj E., Sindi S.H., Tillekeratne L.M.V. (2022). Photoaffinity labeling and bioorthogonal ligation: Two critical tools for designing “Fish Hooks” to scout for target proteins. Bioorg. Med. Chem..

[18] Miyamoto D.K., Flaxman H.A., Wu H.Y., Gao J.X., Woo C.M. (2019). Discovery of a celecoxib binding site on prostaglandin E synthase (PTGES) with a cleavable chelation-assisted biotin probe. ACS Chem. Biol..

[19] Anabuki T., Tsukahara M., Okamoto M., Matsuura H., Takahashi K. (2018). Novel biotin linker with alkyne and amino groups for chemical labelling of a target protein of a bioactive small molecule. Bioorg. Med. Chem. Lett..

[20] Xing M., Wang S., Cui F., Liu H., Gao Z., Ying W., Shi E. (2023). Comprehensive insight on protein modification by V-type agent: A chemical proteomic approach employing bioorthogonal reaction. Proteomics.

[21] Rosenthal A., Rauch S., Eichhorn K.J., Stamma M., Uhlmann P. (2018). Enzyme immobilization on protein-resistant PNIPAAm brushes: Impact of biotin linker length on enzyme amount and catalytic activity. Colloids Surf. B.

[22] Mir F., Shafi S., Zaman M.S., Kalia N.P., Rajput V.S., Mulakayala C., Mulakayala N., Khan I.A., Alam M.S. (2014). Sulfur rich 2-mercaptobenzothiazole and 1,2,3-triazole conjugates as novel antitubercular angent. Eur. J. Med. Chem..

[23] Greene T.W., Wuts P.G.M. (2007). Protective Groups in Organic Synthesis.

