# Supplementary material for: Copper-Catalyzed Azide–Alkyne Cycloaddition-Oriented Multifunctional Bio-Orthogonal Linker BPPA: Design, Synthesis and Evaluation
Source: Molecules. 2023 Dec 14;28(24):8083. doi: 10.3390/molecules28248083 (PMC10745683; doi:10.3390/molecules28248083)
Supplement: Supplementary file 1 [file molecules-28-08083-s001.zip › molecules-2712907-supplementary.pdf]

Supporting Information

CuAAC-oriented multifuctional bioorthogonal linker BPPA: design, synthesis and evaluation

Shuo Wang, Xu He, Junchen Li*, and Enxue Shi*

State Key Laboratory of NBC Protection for Civilian, Beijing 102205, P. R. China

E-mails: exshi@sina.com; lijch07@163.com.

Contents

1. General information 2

2. General procedure for synthesis of 3 2

3. General procedure for synthesis of 4 2

4. Copies of ^{1}H, ^{13}C NMR spectra 3

5. The FTIR spectra of compounds 13

6. The UV/Vis spectra of compounds 18

1. General information

All the commercially available reagents were used without further purification unless otherwise stated. ^{1}H, ^{13}C NMR spectra were recorded at ambient temperature on a Bruker 300 instrument. All the spectra were referenced to $CDCl_3$ (^{1}H δ 7.26 ppm and ^{13}C NMR δ 77.00 ppm). Data were reported as follows: chemical shift, multiplicity (s = singlet, d = doublet, t = triplet, q = quartet, dd = doublet of doublets, td = triplet of doublets, qd = quartet of doublets, m = multiplet), coupling constants (Hz) and integration. High-resolution mass spectra (HRMS) were obtained on an Agilent 6545 Q-TOF HPLC and mass spectrometry. FTIR spectra were recorded on a Bruker VERTEX70 Tango-R spectrophotometer. UV-Vis spectra were conducted on a Shimadzu UV-2550 spectrophotometer.

2. General procedure for synthesis of 3

To a solution of Biotin-PEG$_n$-COOH **1** (0.2 mmol) and K_2CO_3 (0.1 mmol) in H_2O (1 ml) was added a solution of 4-ethynylbenzoyl bromide (0.2 mmol) or 4-azidobenzoyl bromide (0.2 mmol) **2** in EtOH (1 ml) under air atmosphere. After stirring at 50 °C for 4 h, column chromatography separation (DCM: CH_3OH = 10:1) gave the target products **3**.

3. General procedure for synthesis of 4

To a solution of **3a** (0.2 mmol) and 3,3-dimethylbut-1-yne (0.2 mmol) in EtOH (1 ml) was added a solution of natrascorb (0.04 mmol) and $CuSO_4$ (0.02 mmol) in H_2O (1 ml). After stirring at ambient temperature for 1 h, **4a** was obtained as a white solid after HPLC purification using a C8 (30 x 250 mm) column with CH_3CN/H_2O (35/65) as mobile phase.

To a solution of **3e** (0.2 mmol) and 2-azidopropane (0.2 mmol) in EtOH (1 ml) was added a solution of natrascorb (0.04 mmol) and $CuSO_4$ (0.02 mmol) in H_2O (1 ml). After stirring at ambient temperature for 1 h, **4b** was obtained as a white solid after HPLC purification using a C8 (30 x 250 mm) column with CH_3CN/H_2O (35/65) as mobile phase.

4. Copies of ^{1}H, ^{13}C NMR spectra

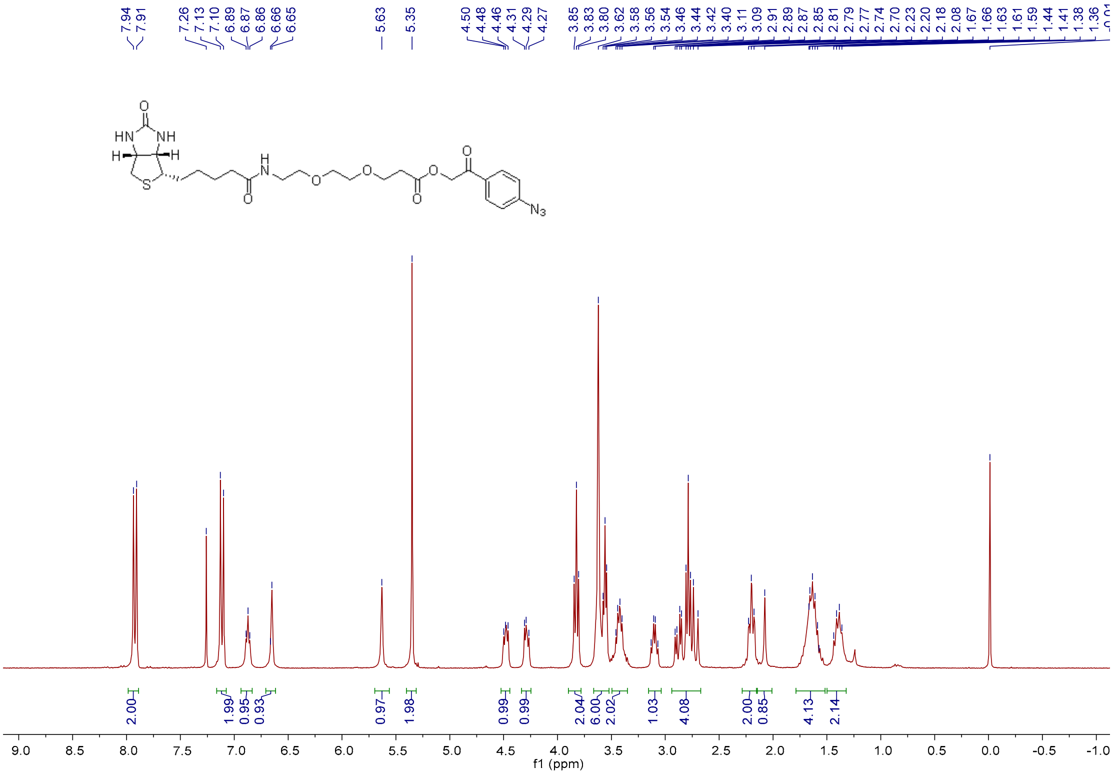

Figure S1. ^{1}H NMR of compound **3a**

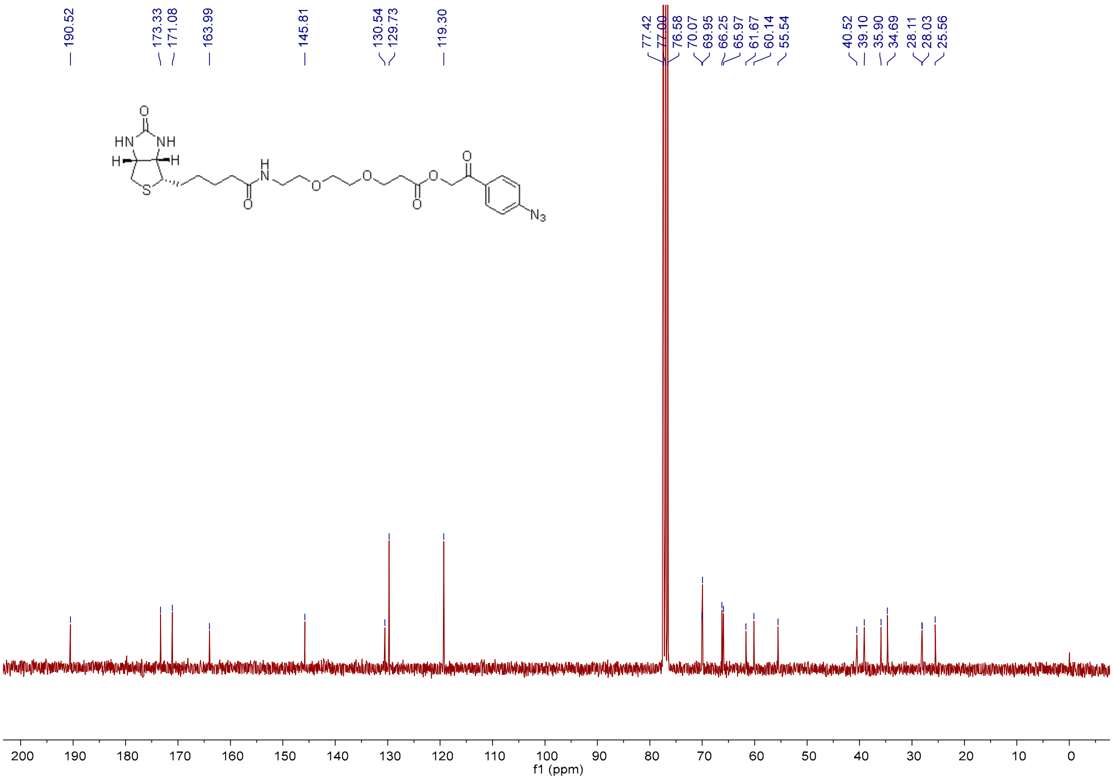

Figure S2. ^{13}C NMR of compound **3a**

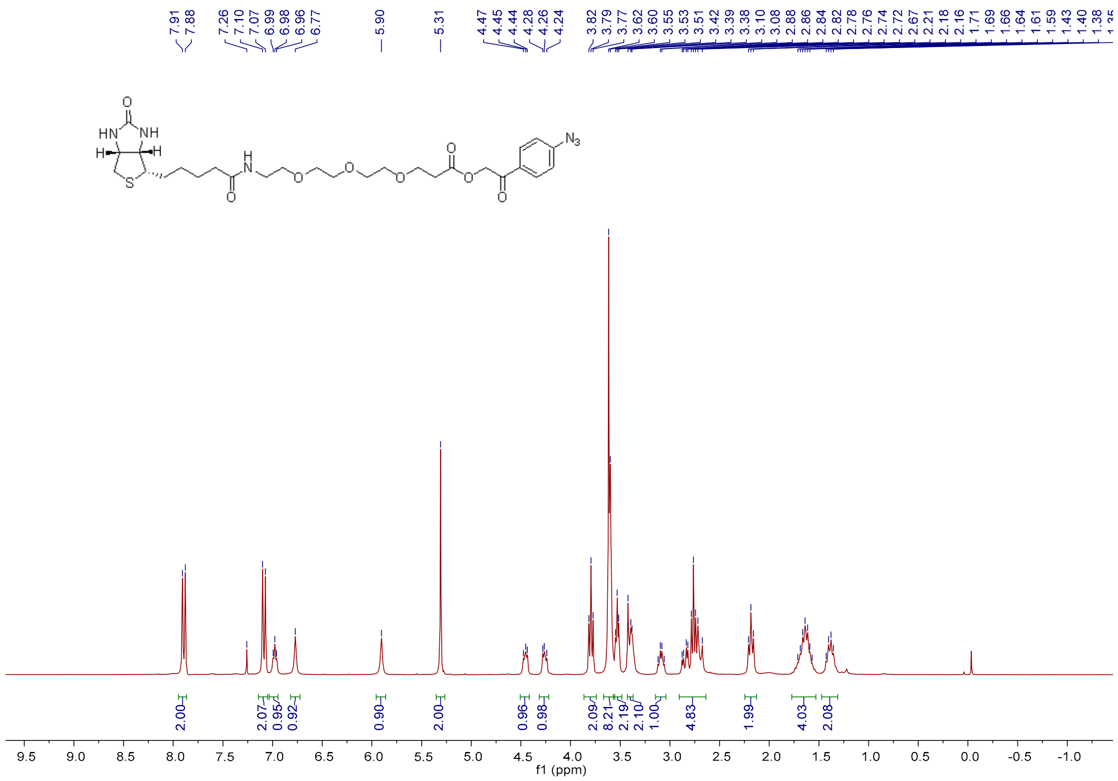

Figure S3. ^{1}H NMR of compound **3b**

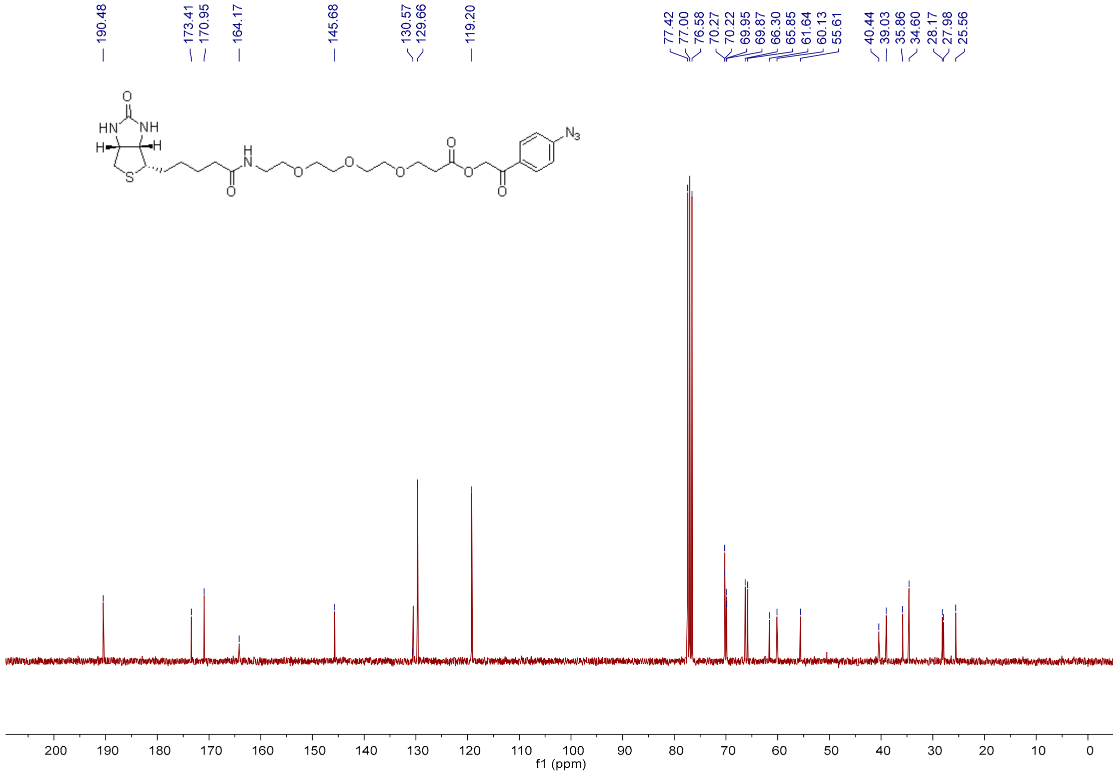

Figure S4. ^{13}C NMR of compound **3b**

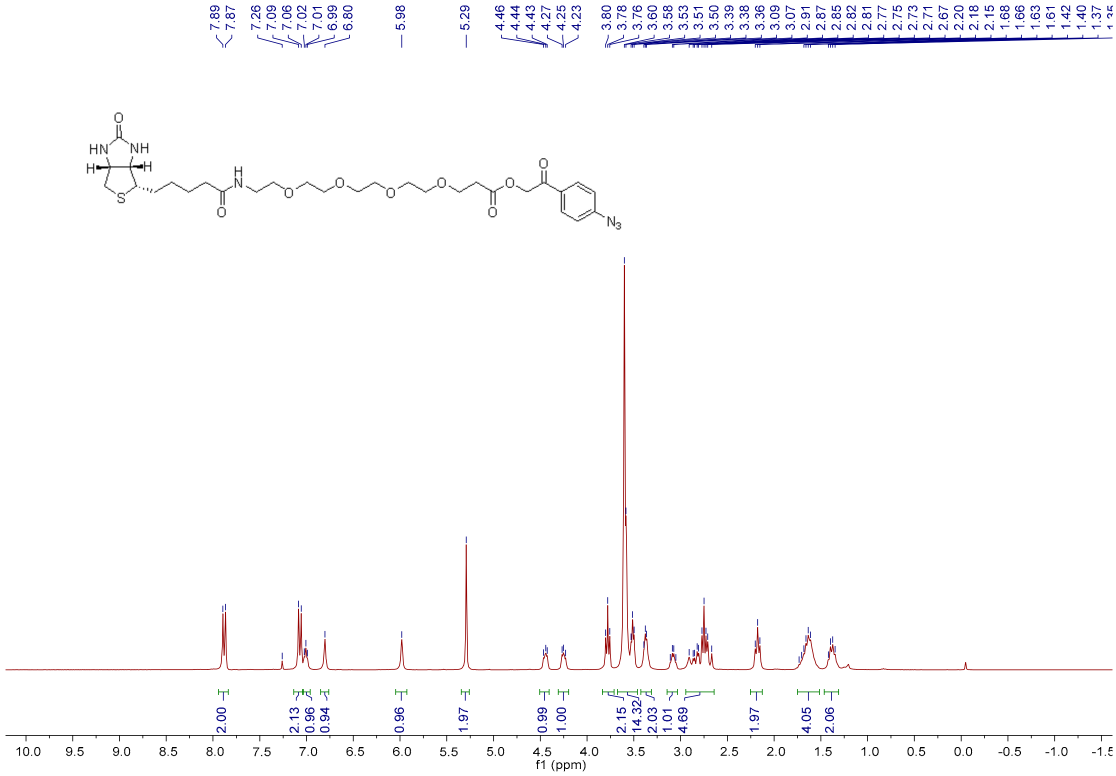

Figure S5. ^{1}H NMR of compound **3c**

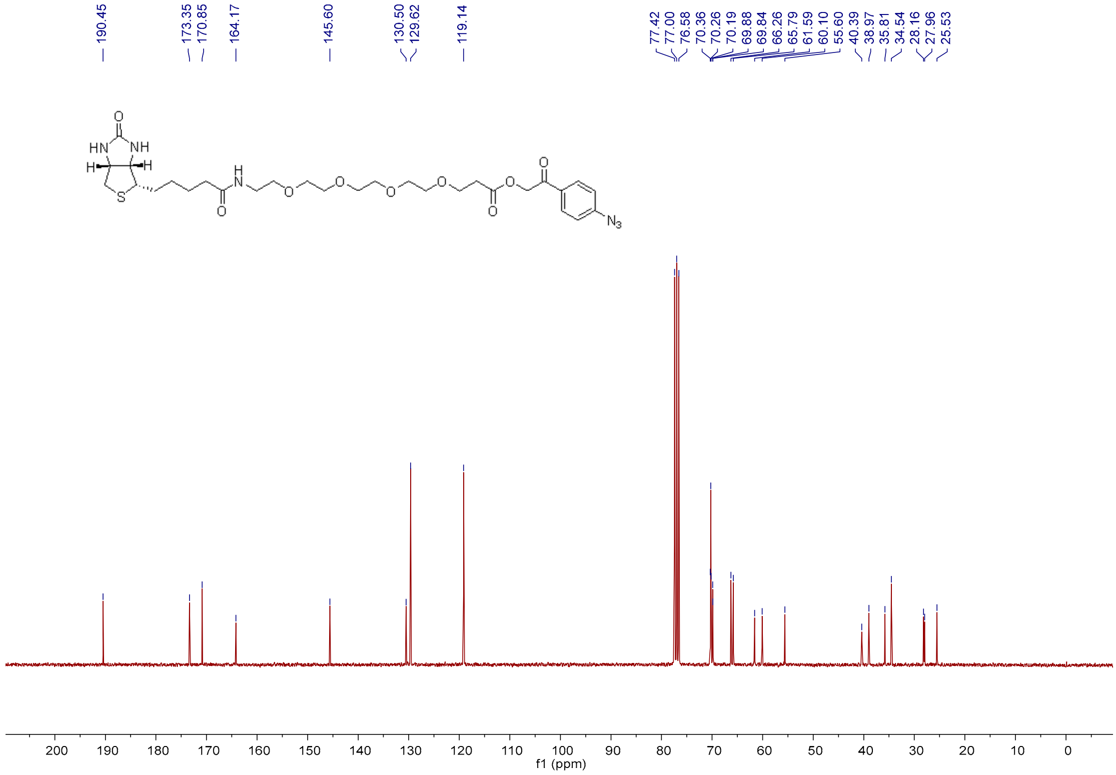

Figure S6. ^{13}C NMR of compound **3c**

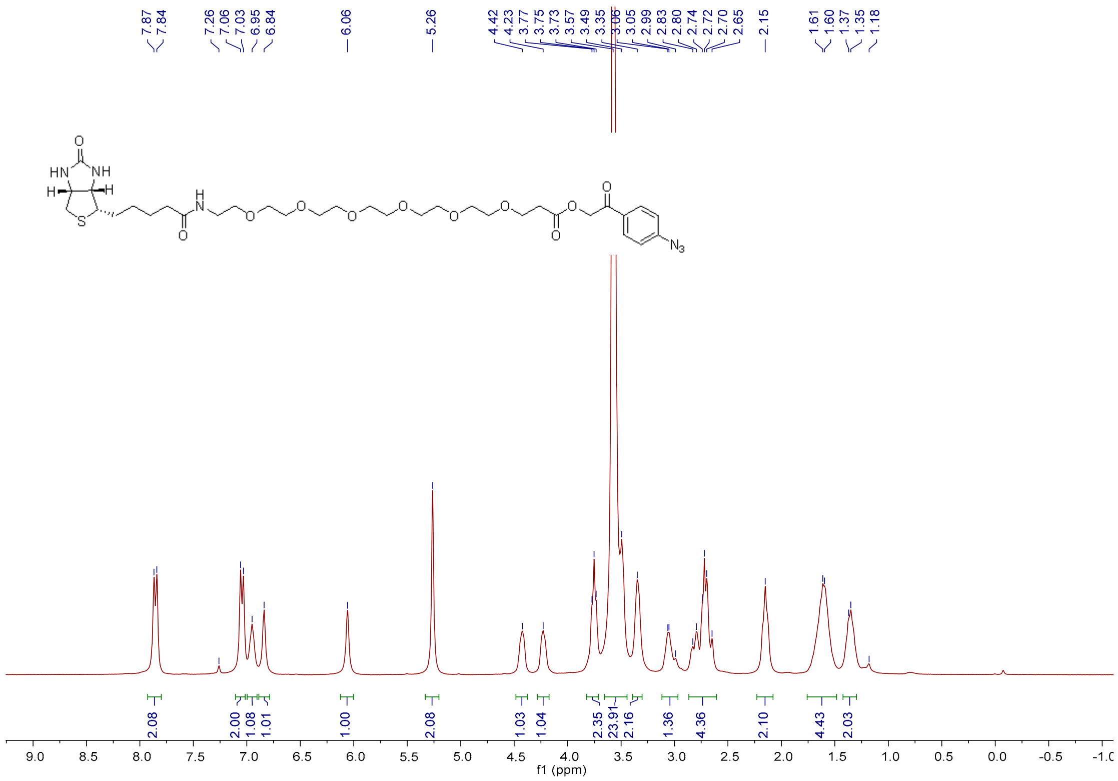

Figure S7. ^{1}H NMR of compound **3d**

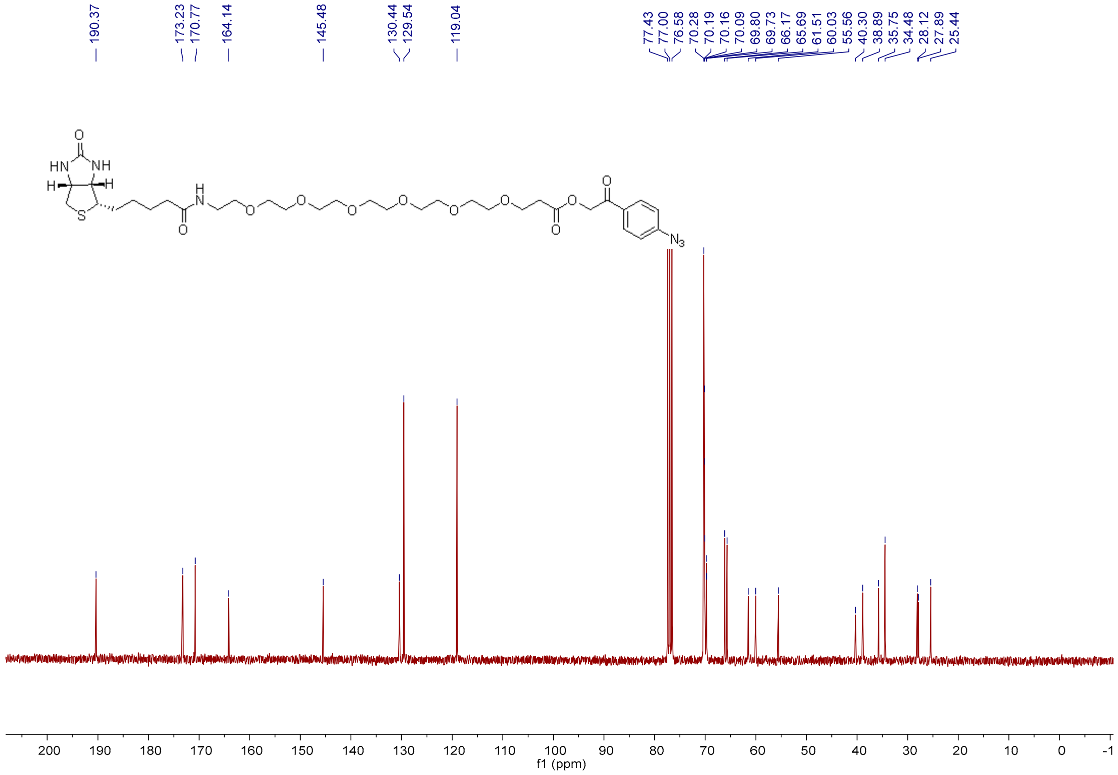

Figure S8. ^{13}C NMR of compound **3d**

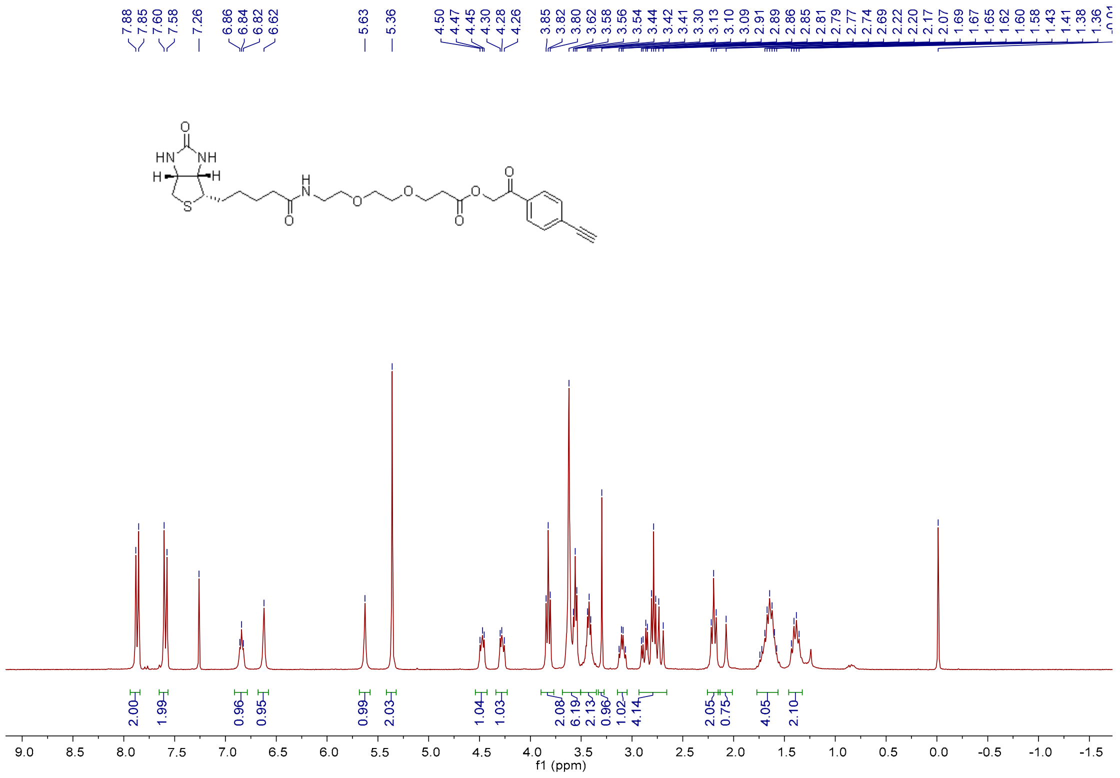

Figure S9. ^{1}H NMR of compound **3e**

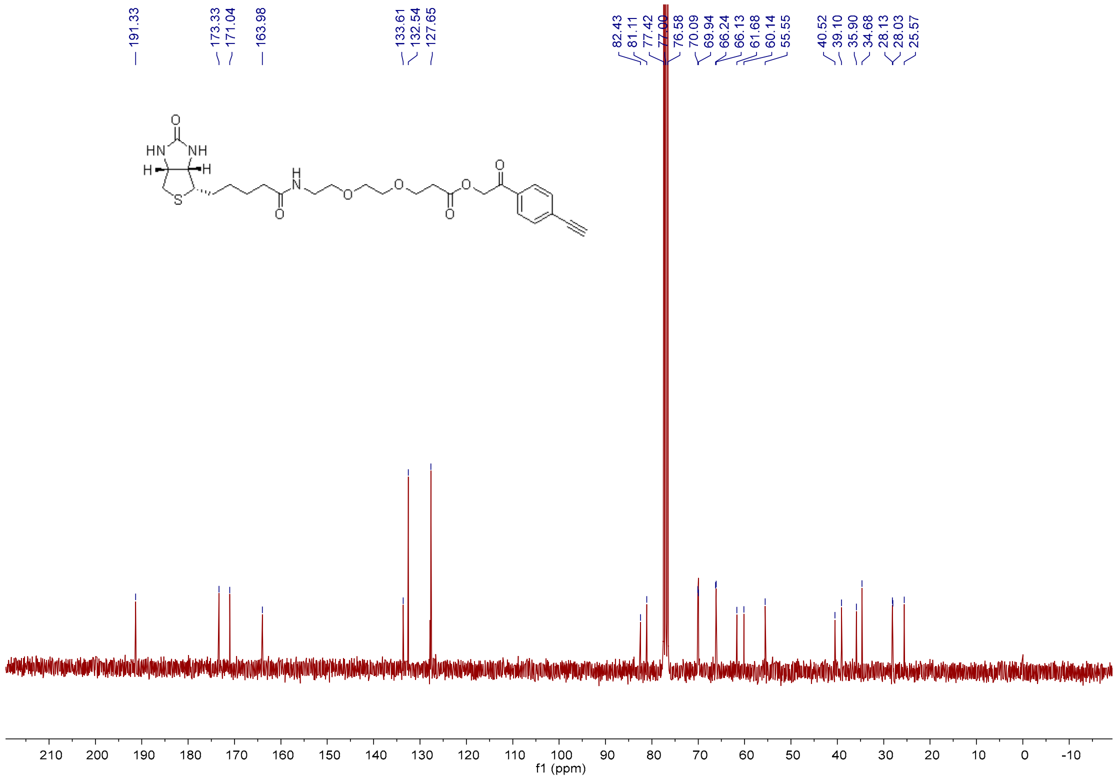

Figure S10. ^{13}C NMR of compound **3e**

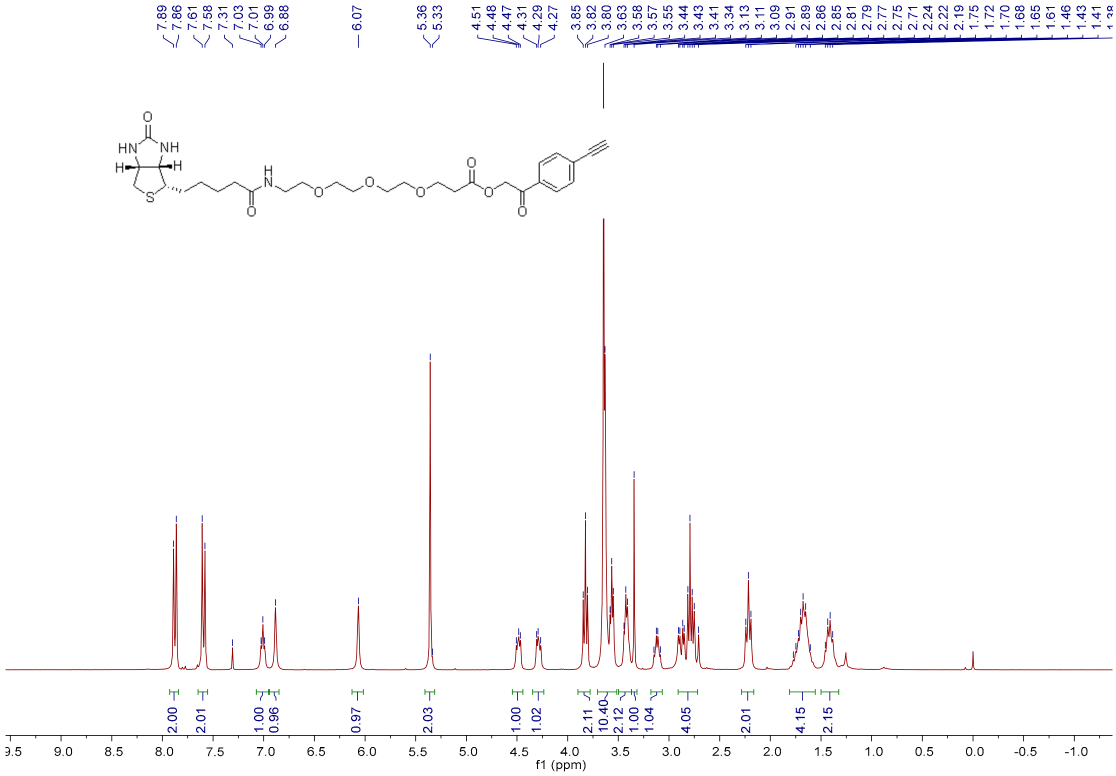

Figure S11. ^{1}H NMR of compound **3f**

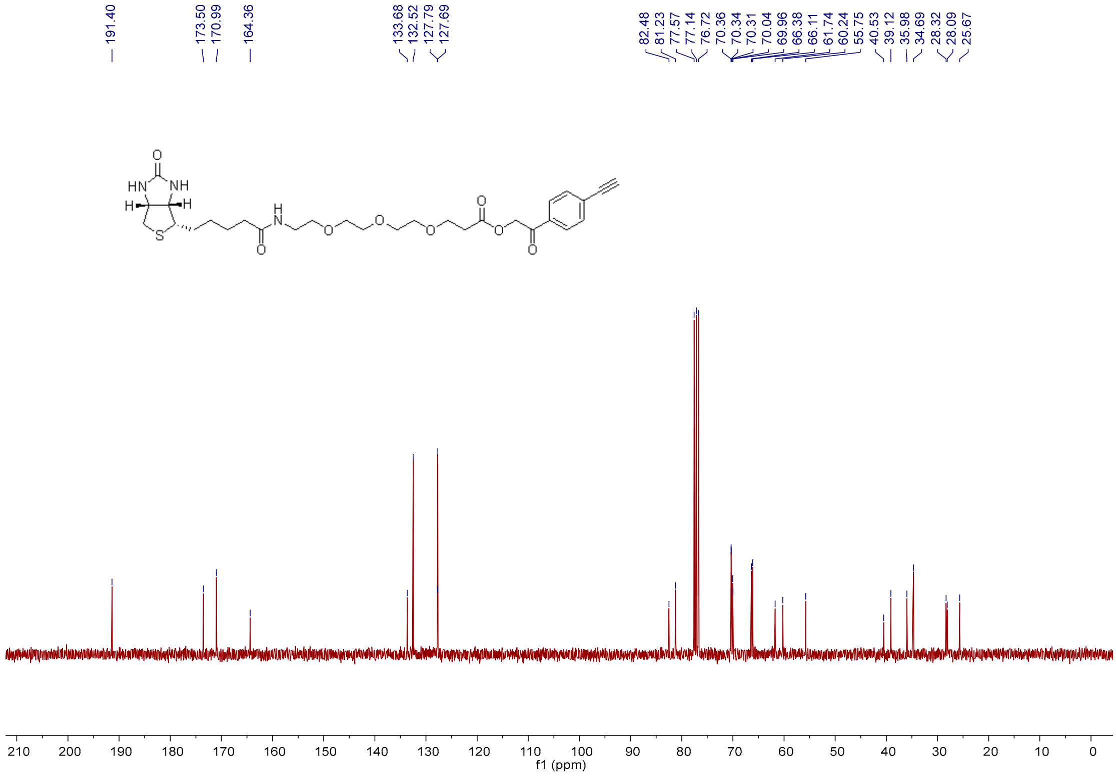

Figure S12. ^{13}C NMR of compound **3f**

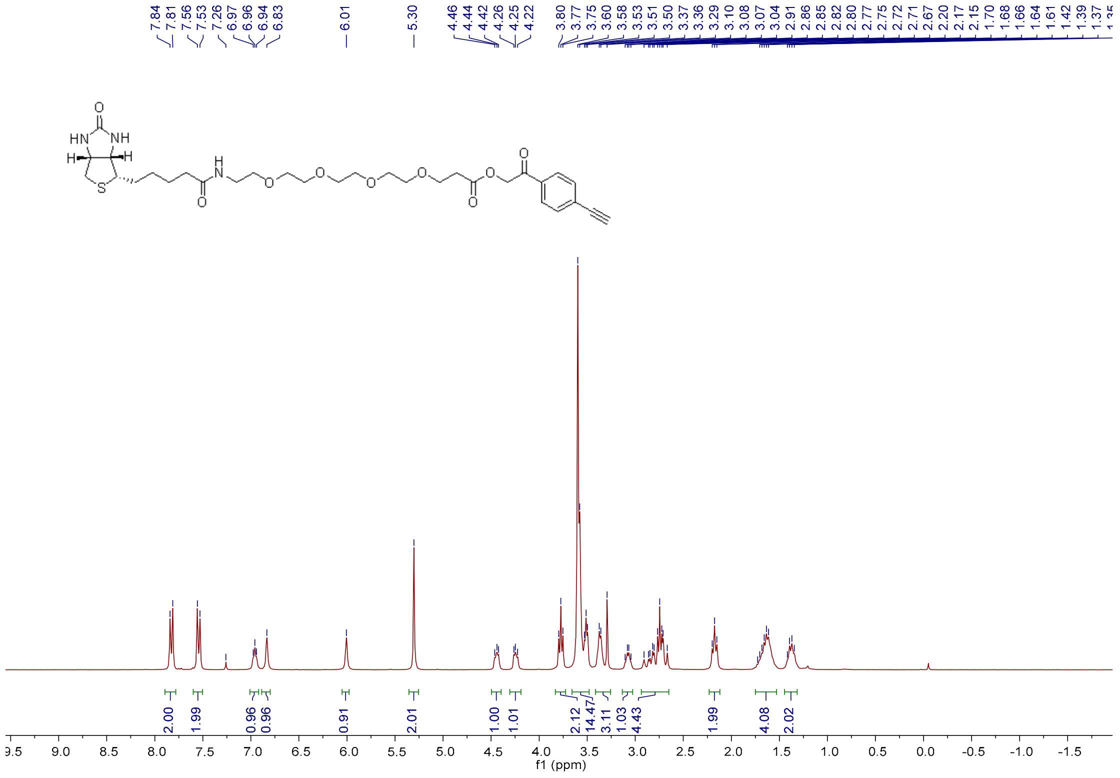

Figure S13. ^{1}H NMR of compound **3g**

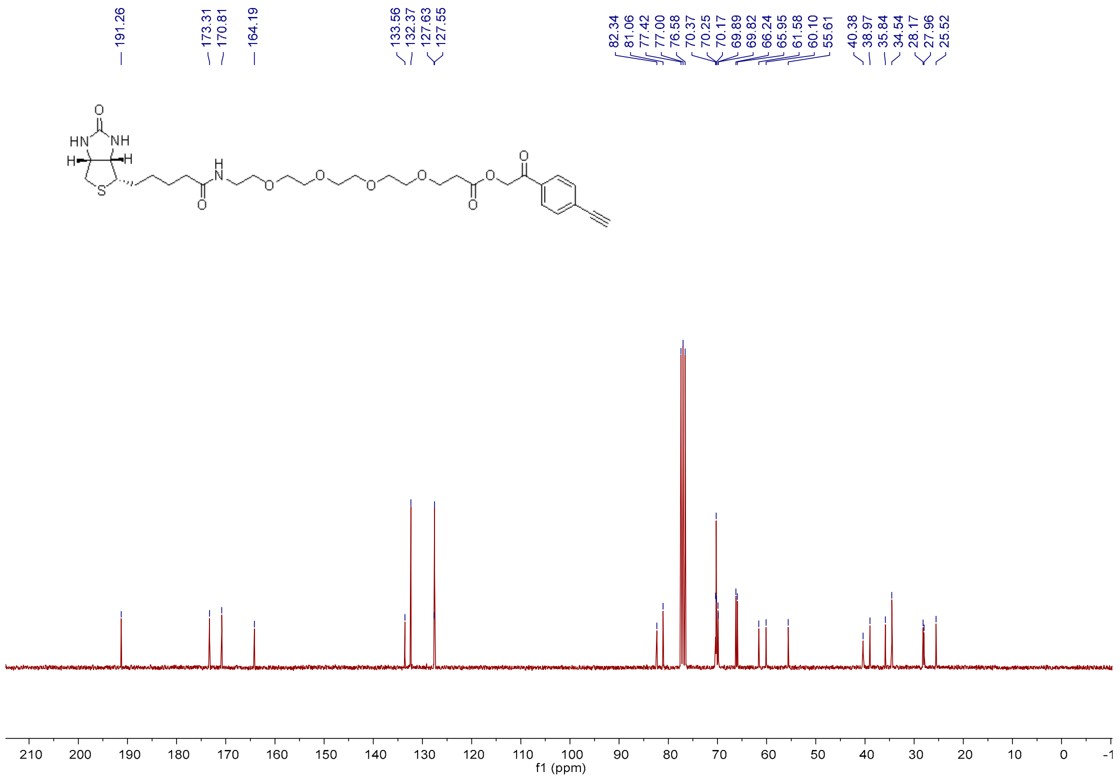

Figure S14. ^{13}C NMR of compound **3g**

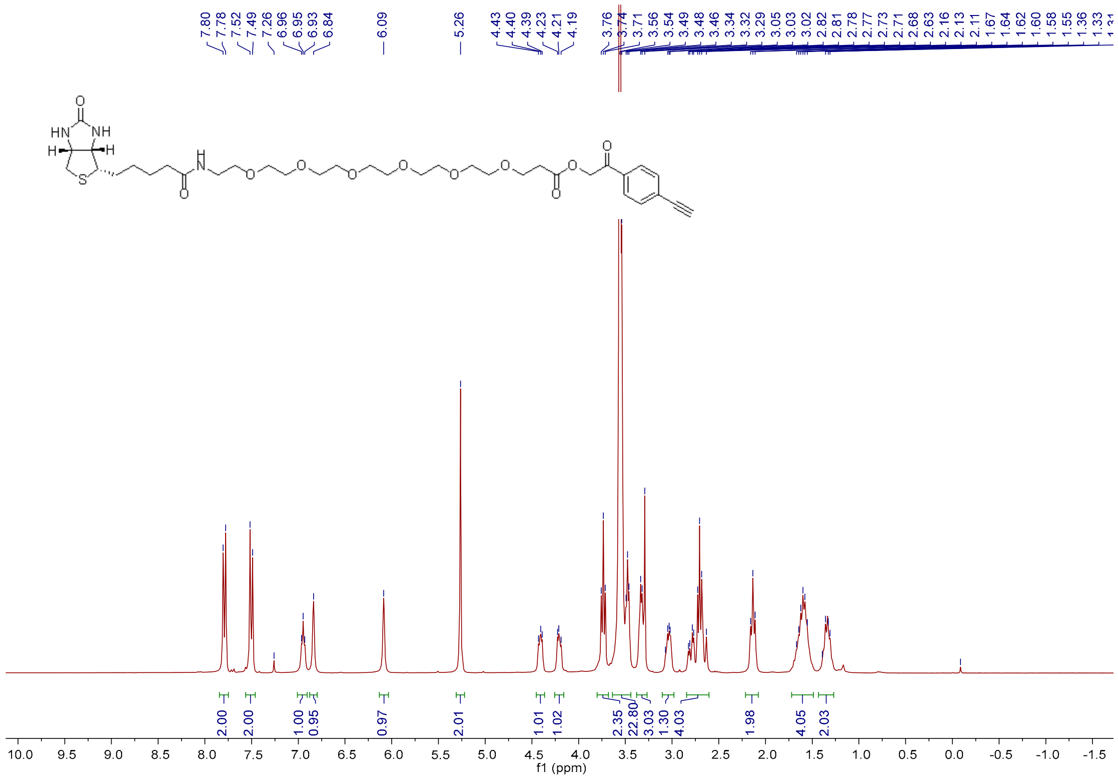

Figure S15. ^{1}H NMR of compound **3h**

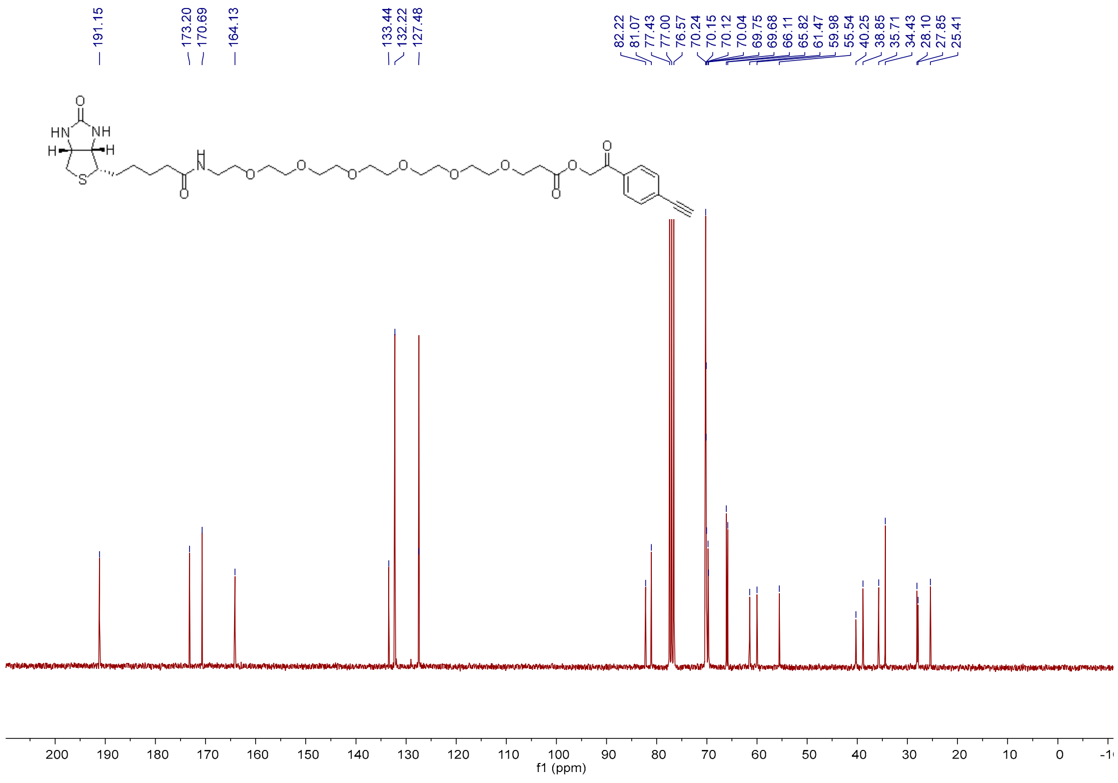

Figure S16. ^{13}C NMR of compound **3h**

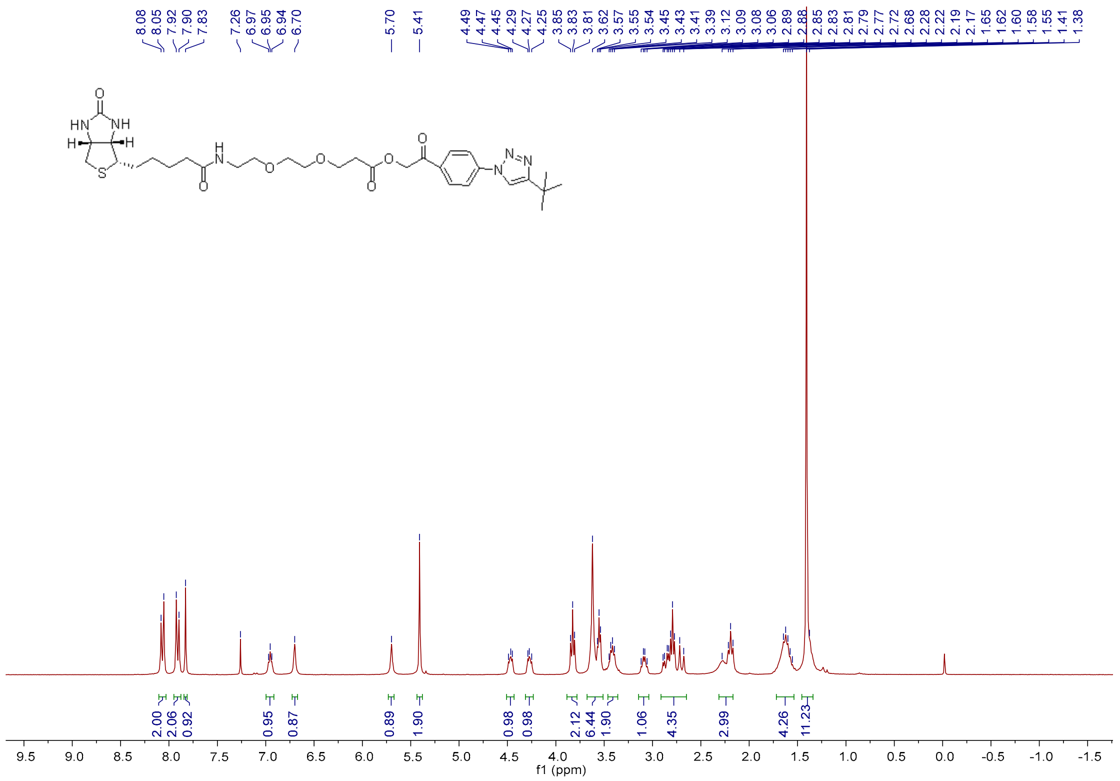

Figure S17. ^{1}H NMR of compound **4a**

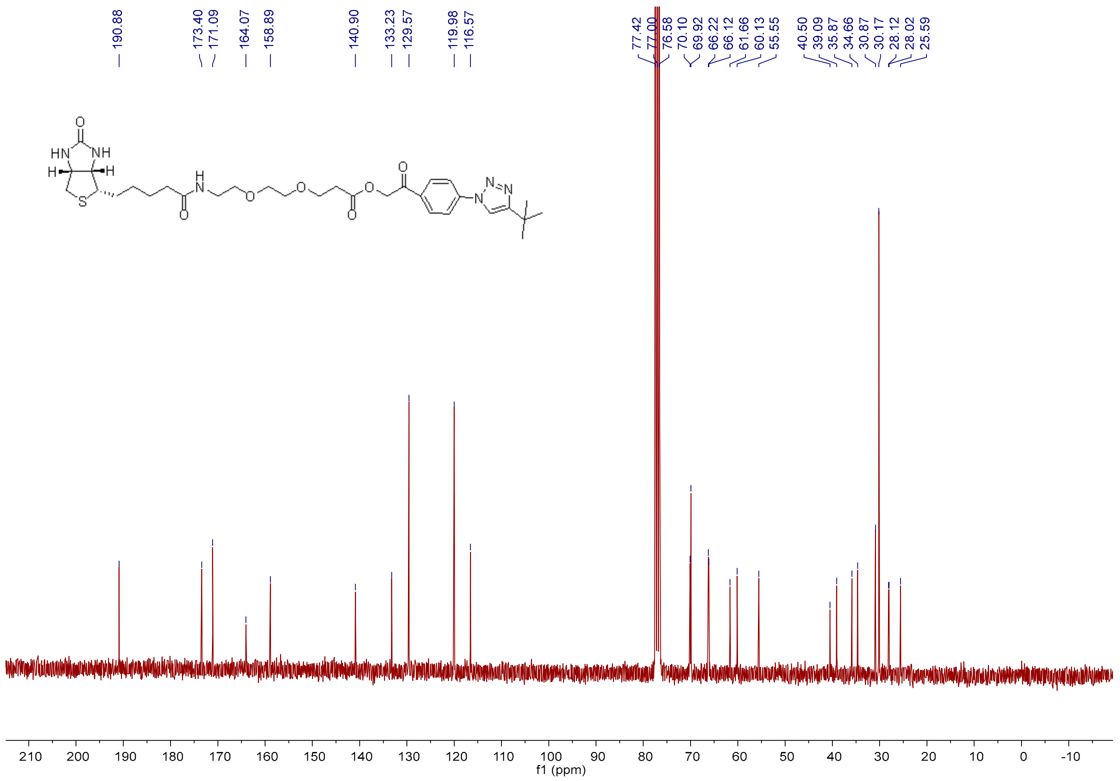

Figure S18. ^{13}C NMR of compound **4a**

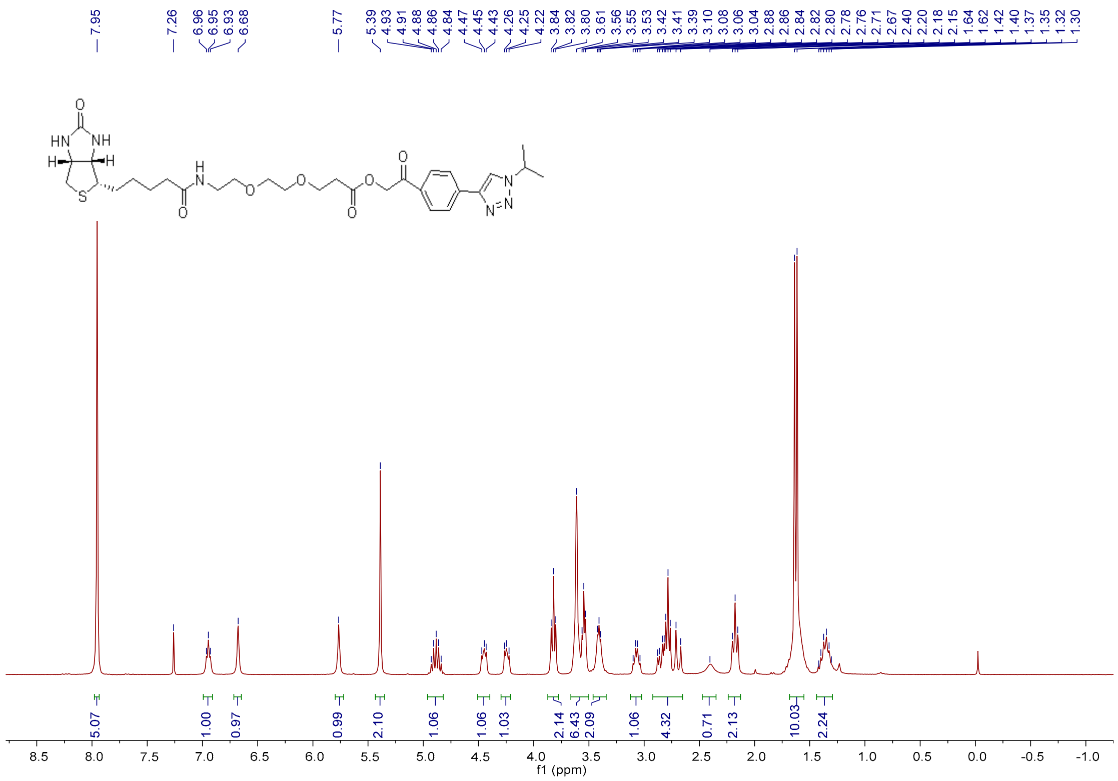

Figure S19. ^{1}H NMR of compound **4b**

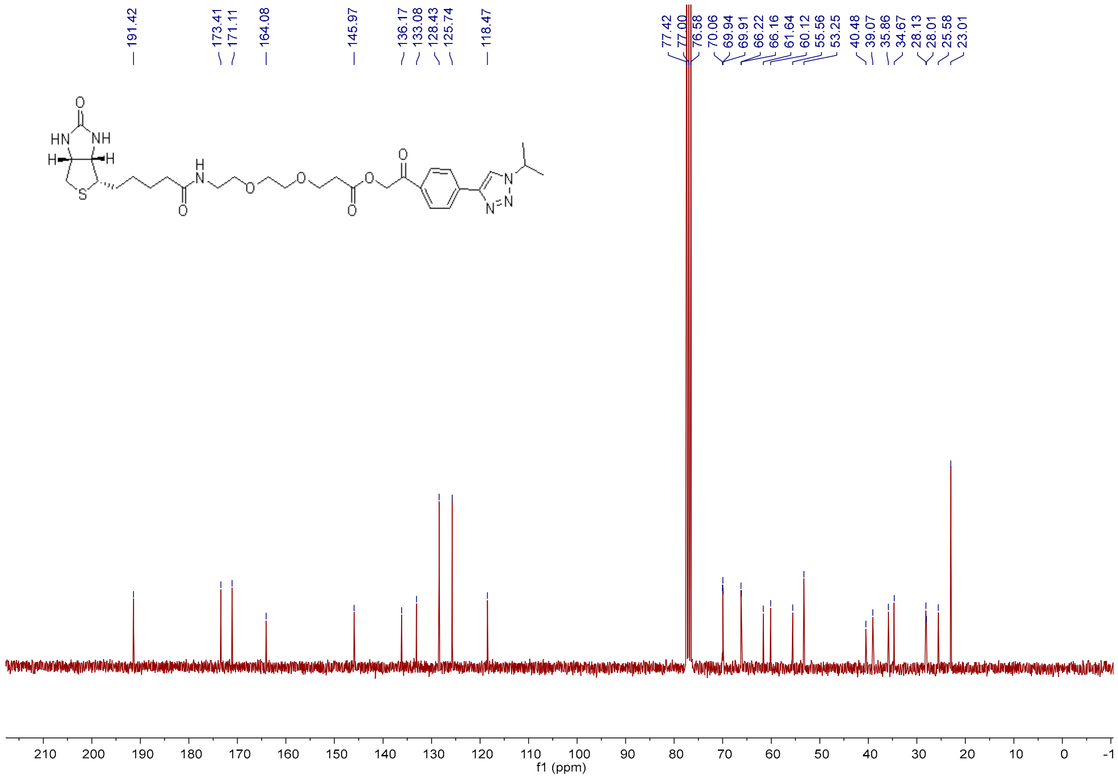

Figure S20. ^{13}C NMR of compound **4b**

5. The FTIR spectra of compounds

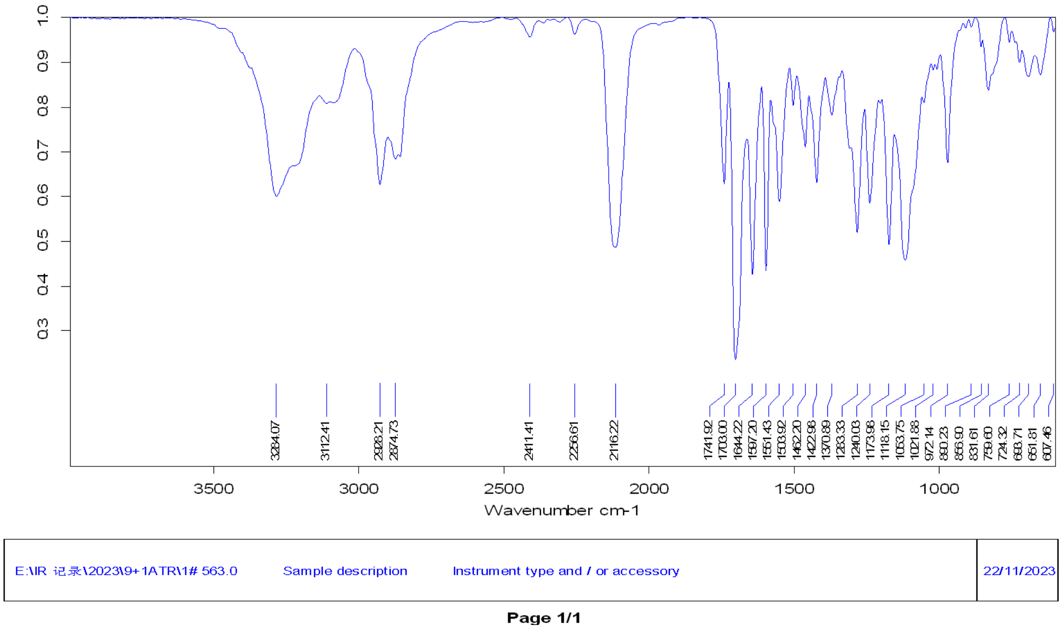

Figure S21. The FTIR spectra of compound **3a**

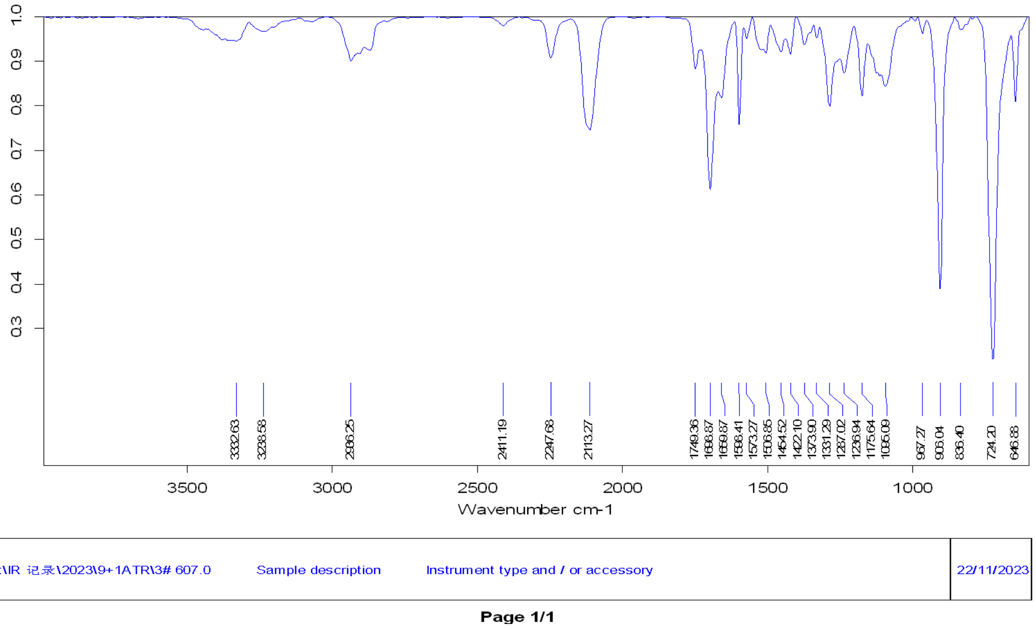

Figure S22. The FTIR spectra of compound **3b**

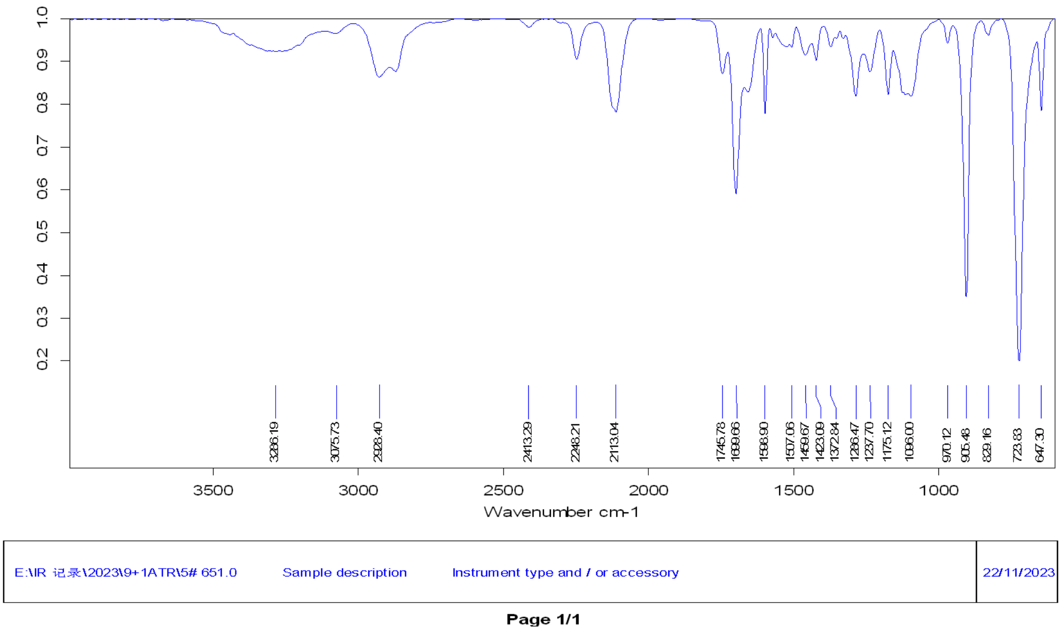

Figure S23. The FTIR spectra of compound **3c**

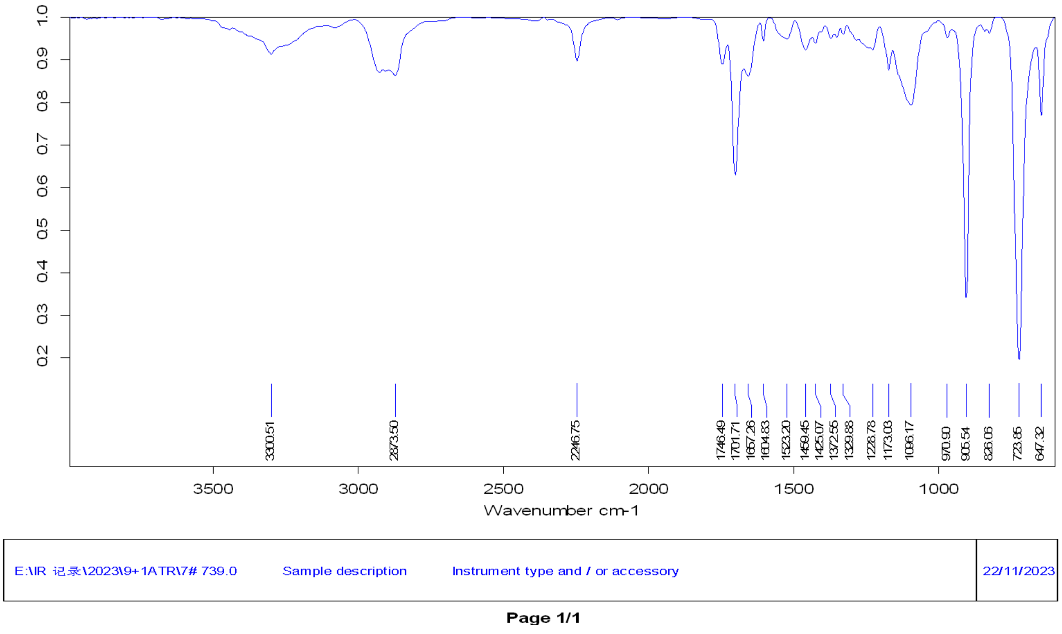

Figure S24. The FTIR spectra of compound **3d**

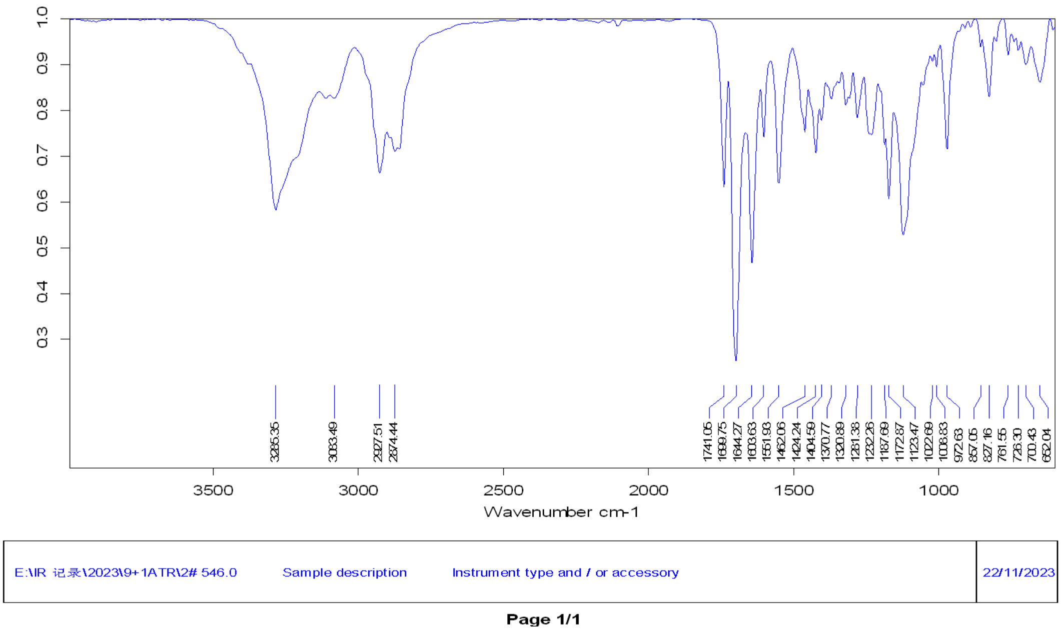

Figure S25. The FTIR spectra of compound **3e**

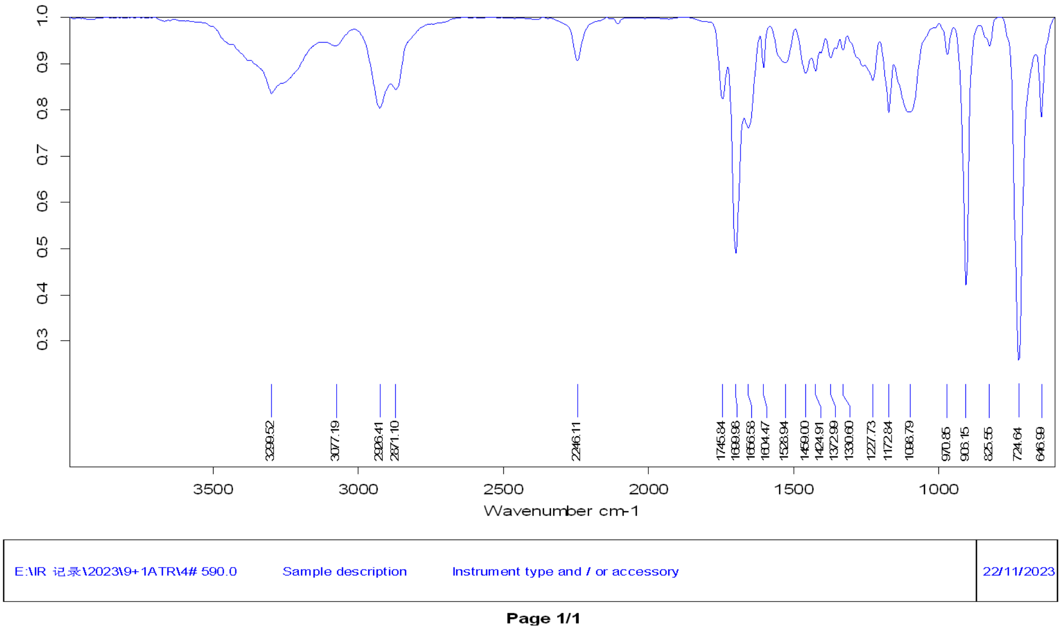

Figure S26. The FTIR spectra of compound **3f**

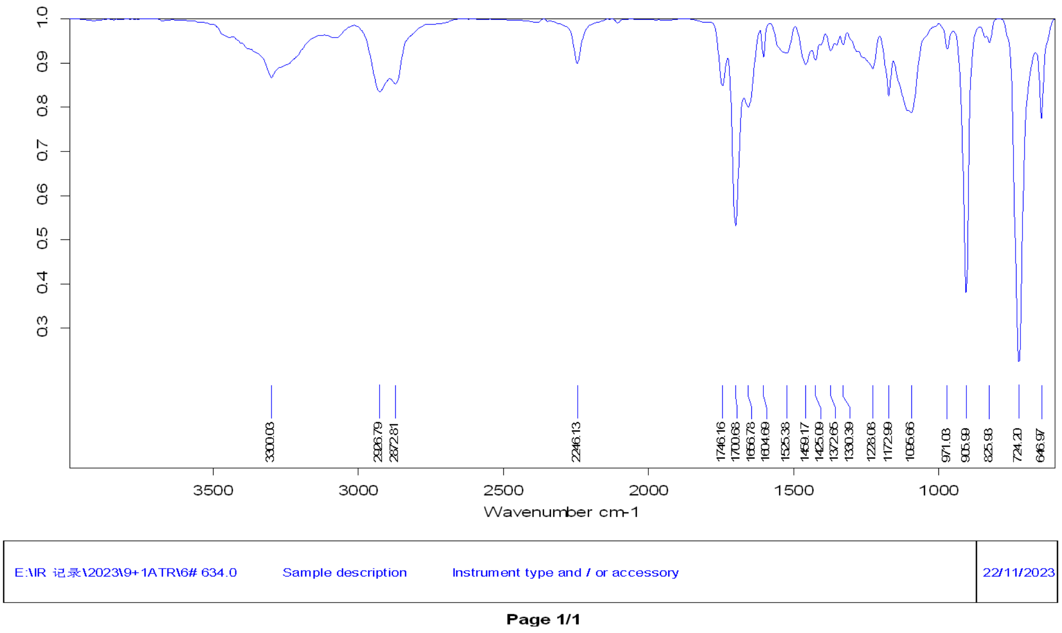

Figure S27. The FTIR spectra of compound **3g**

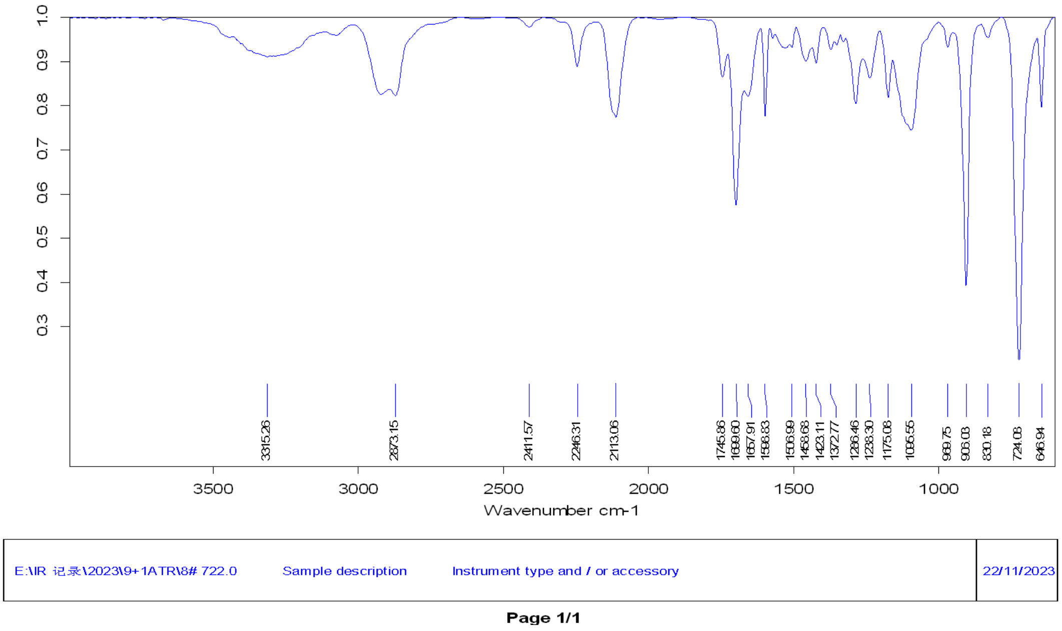

Figure S28. The FTIR spectra of compound **3h**

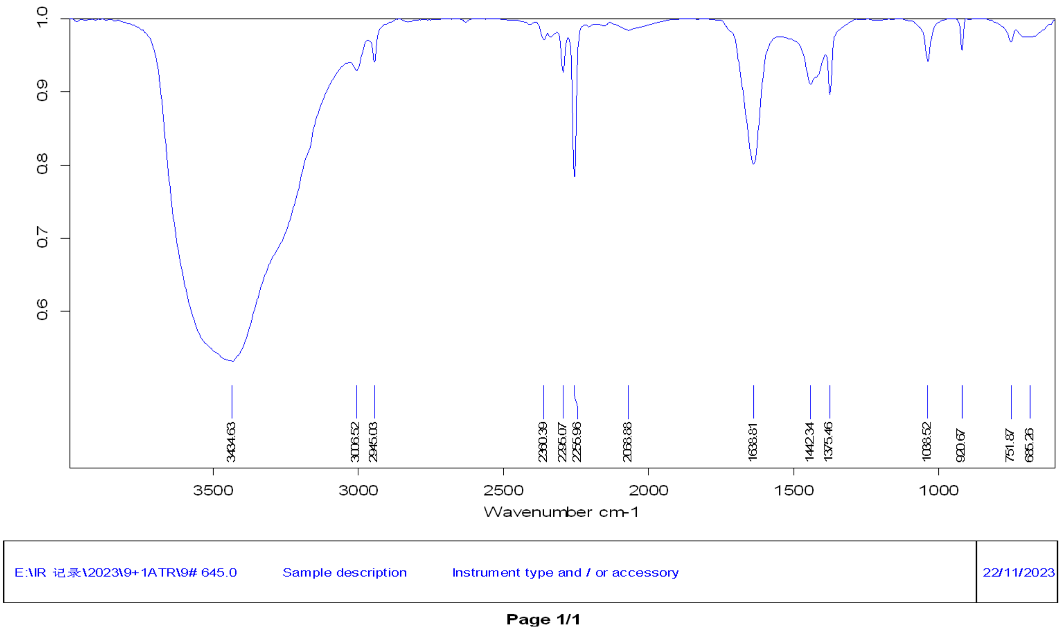

Figure S29. The FTIR spectra of compound **4a**

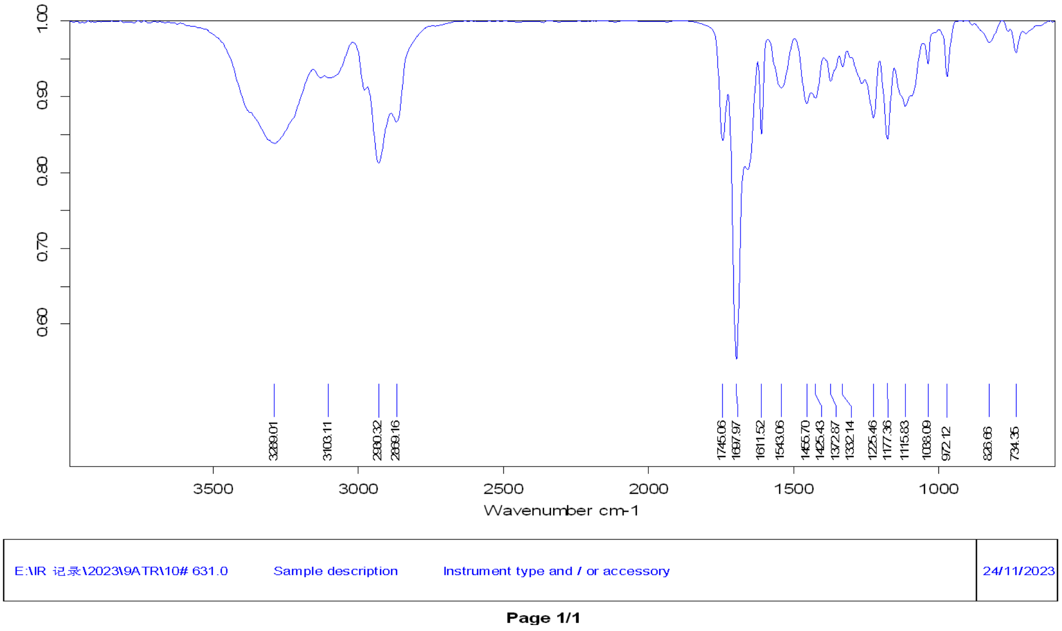

Figure S30. The FTIR spectra of compound **4b**

6. The UV/Vis spectra of compounds

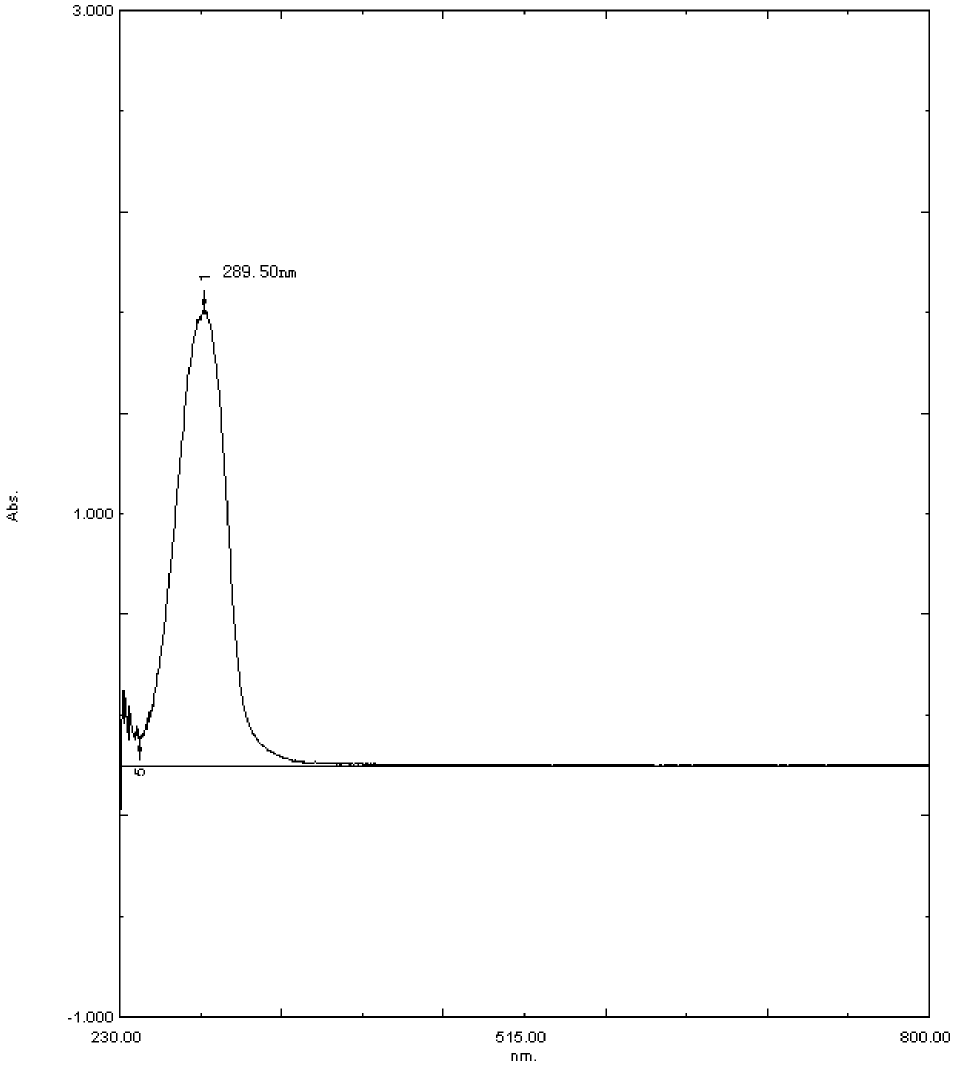

Figure S31. The UV/Vis spectra of compound **3a**

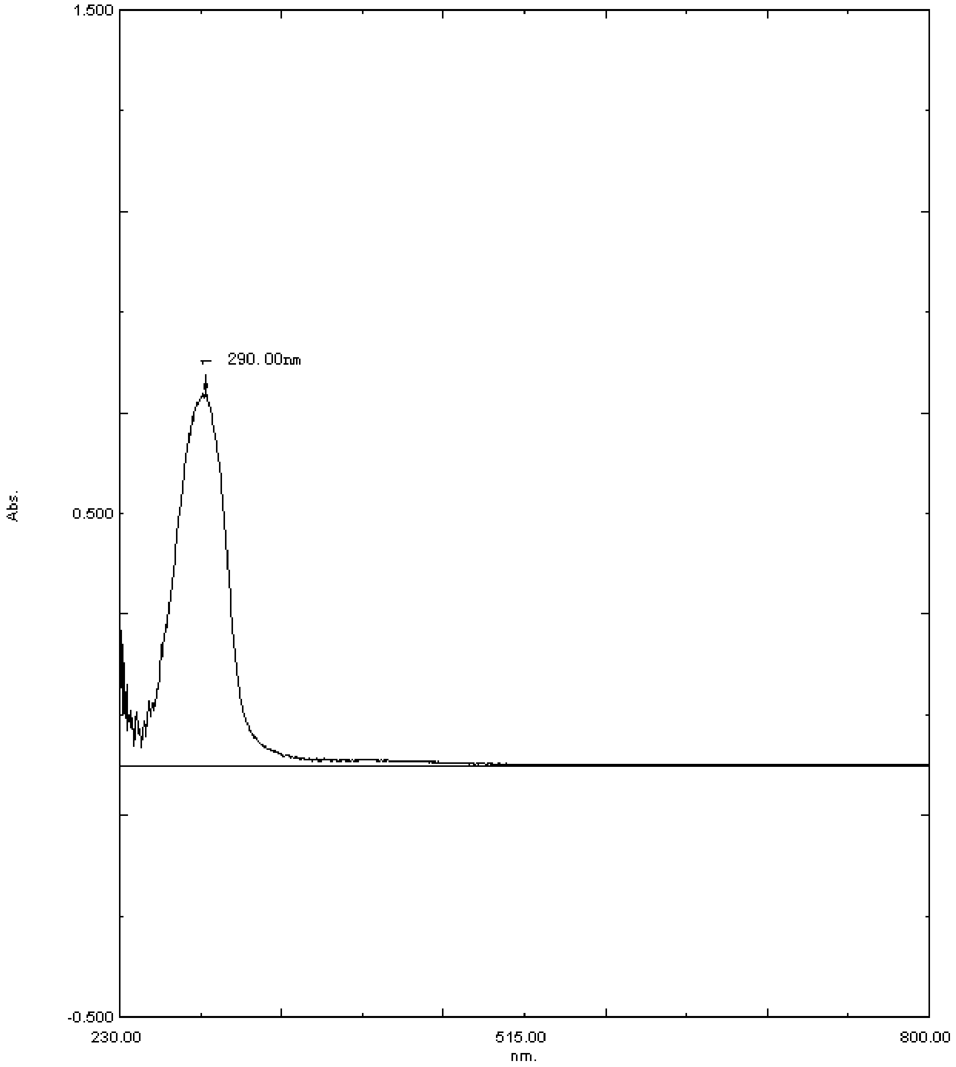

Figure S32. The UV/Vis spectra of compound **3b**

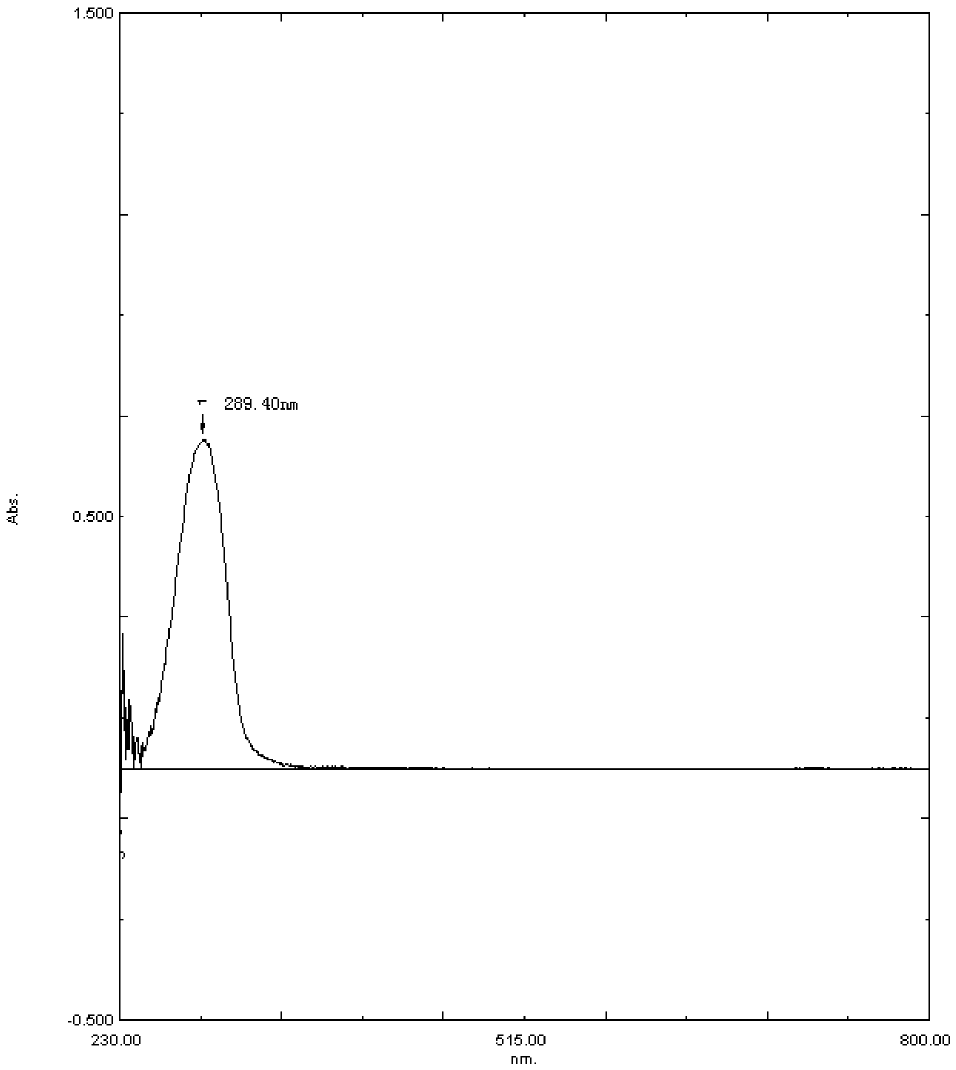

Figure S33. The UV/Vis spectra of compound **3c**

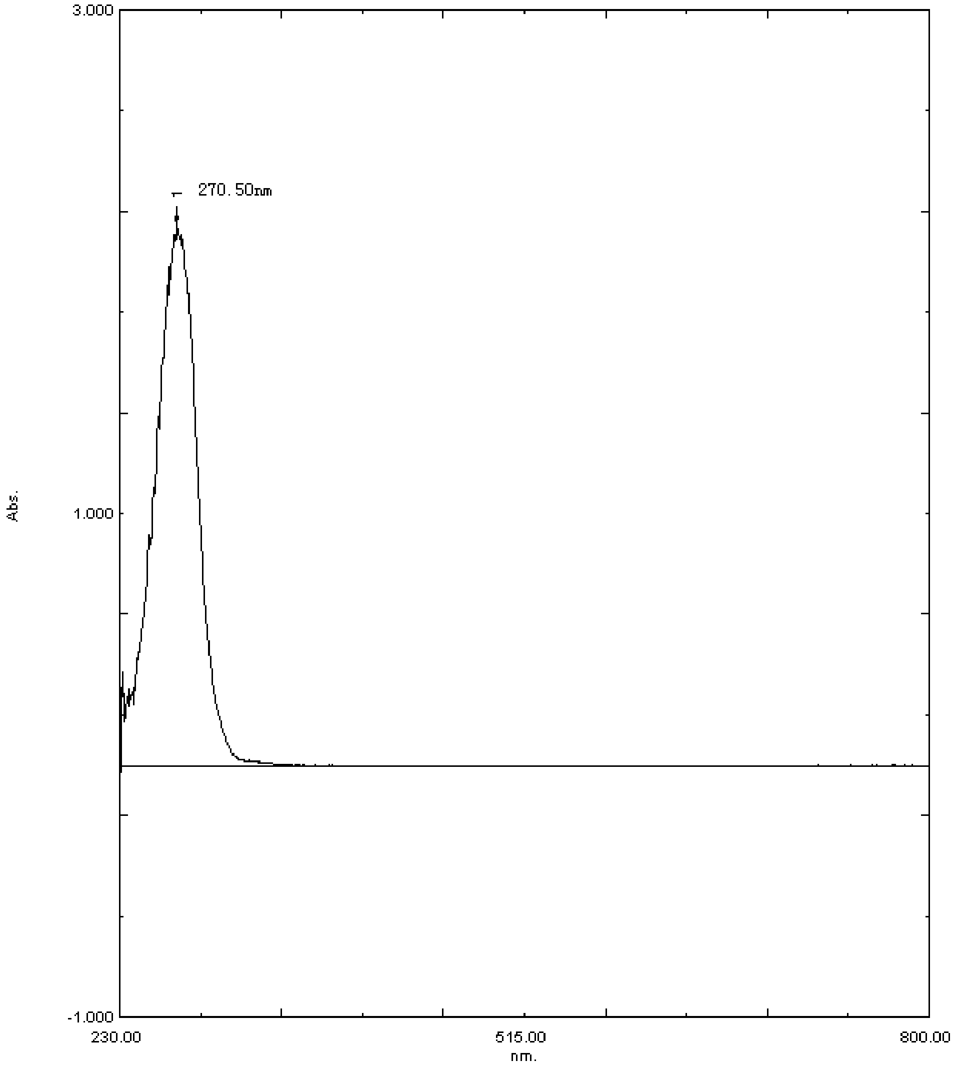

Figure S34. The UV/Vis spectra of compound **3d**

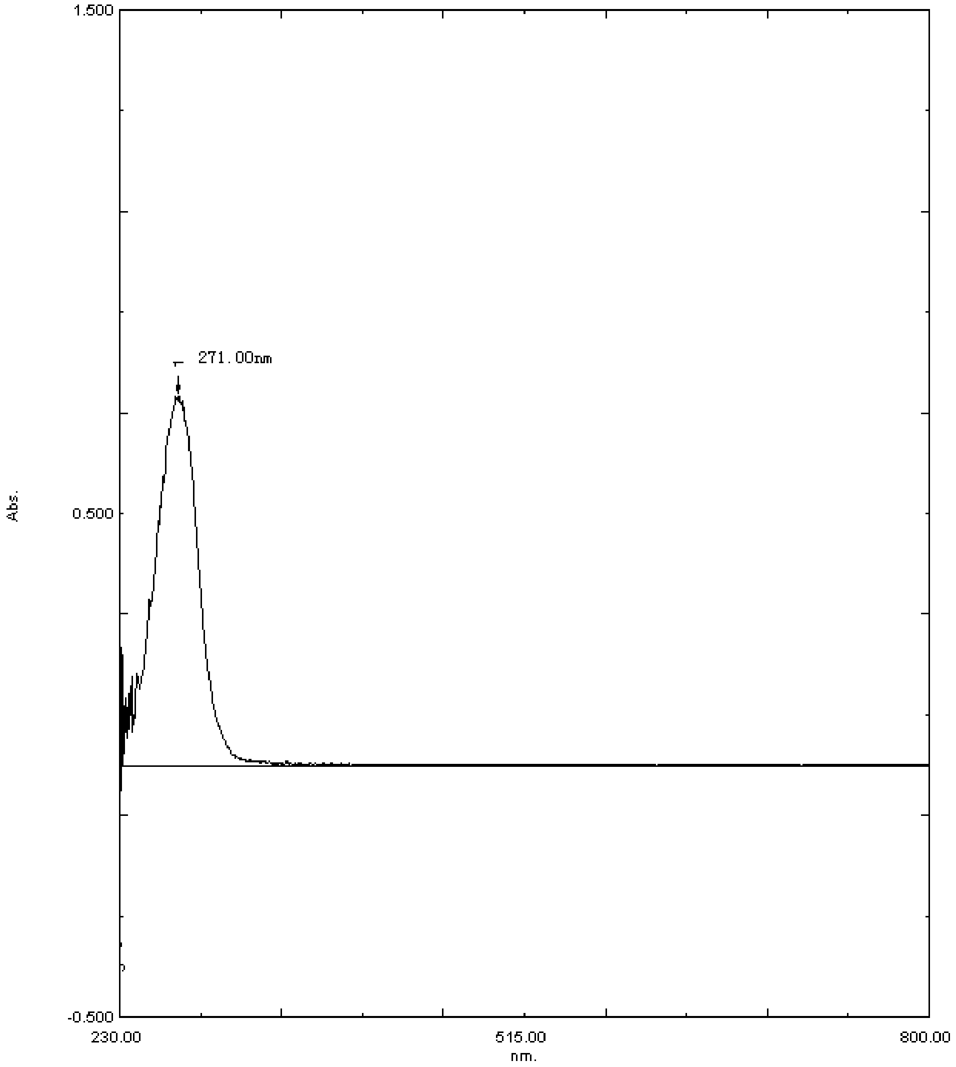

Figure S35. The UV/Vis spectra of compound **3e**

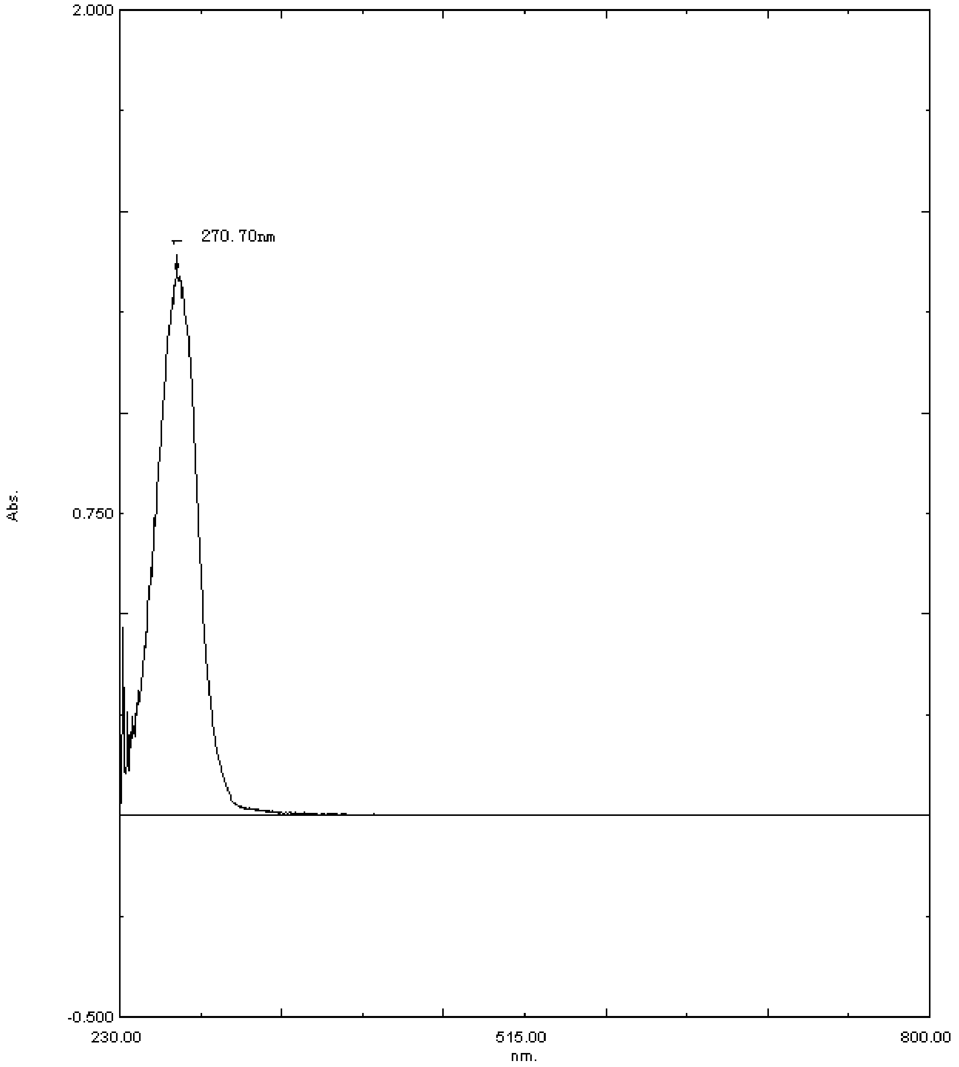

Figure S36. The UV/Vis spectra of compound **3f**

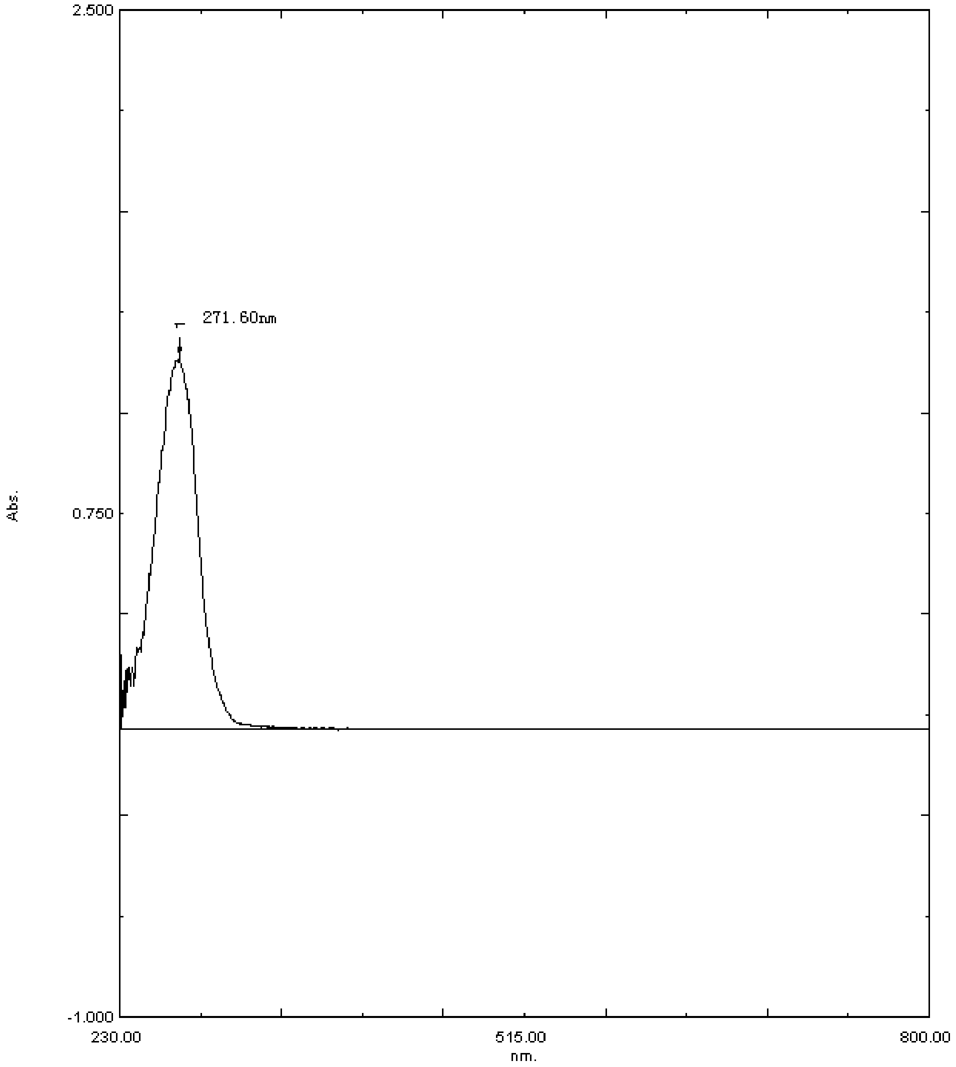

Figure S37. The UV/Vis spectra of compound **3g**

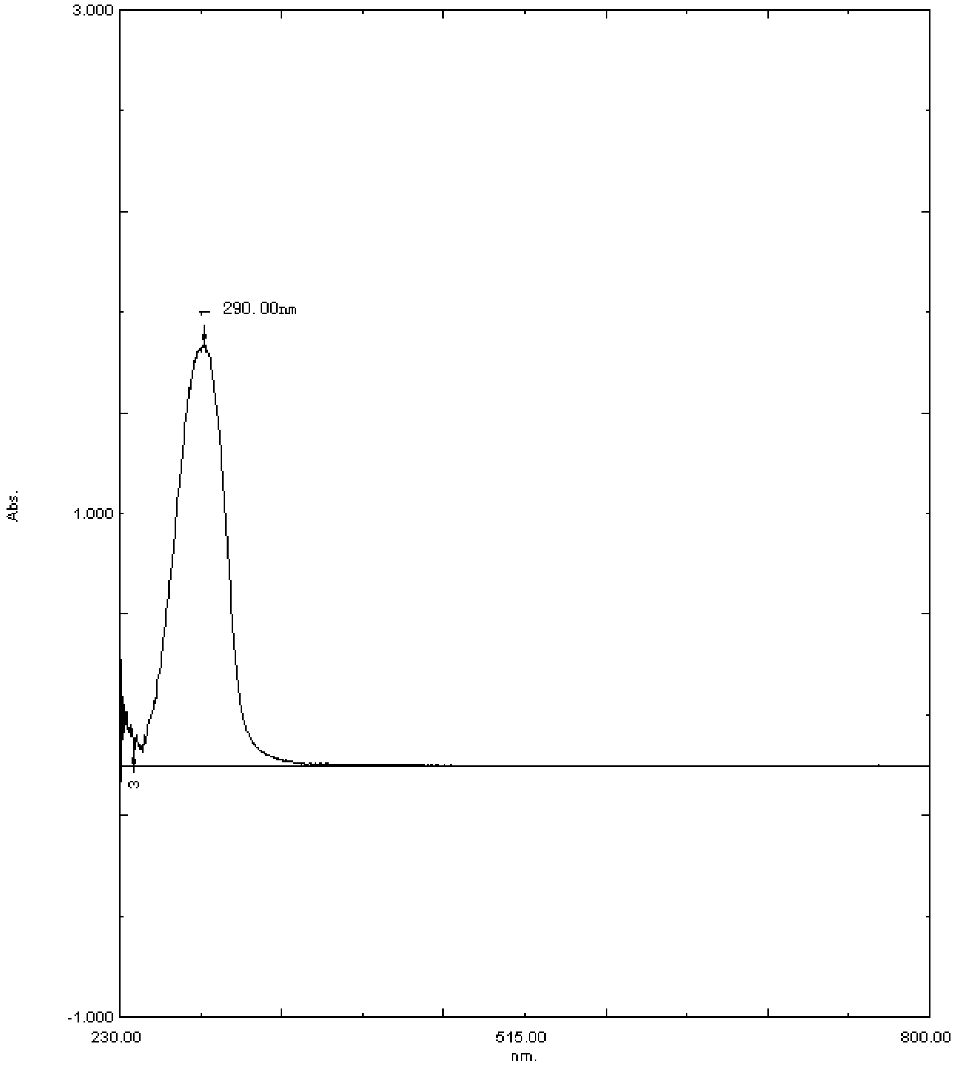

Figure S38. The UV/Vis spectra of compound **3h**

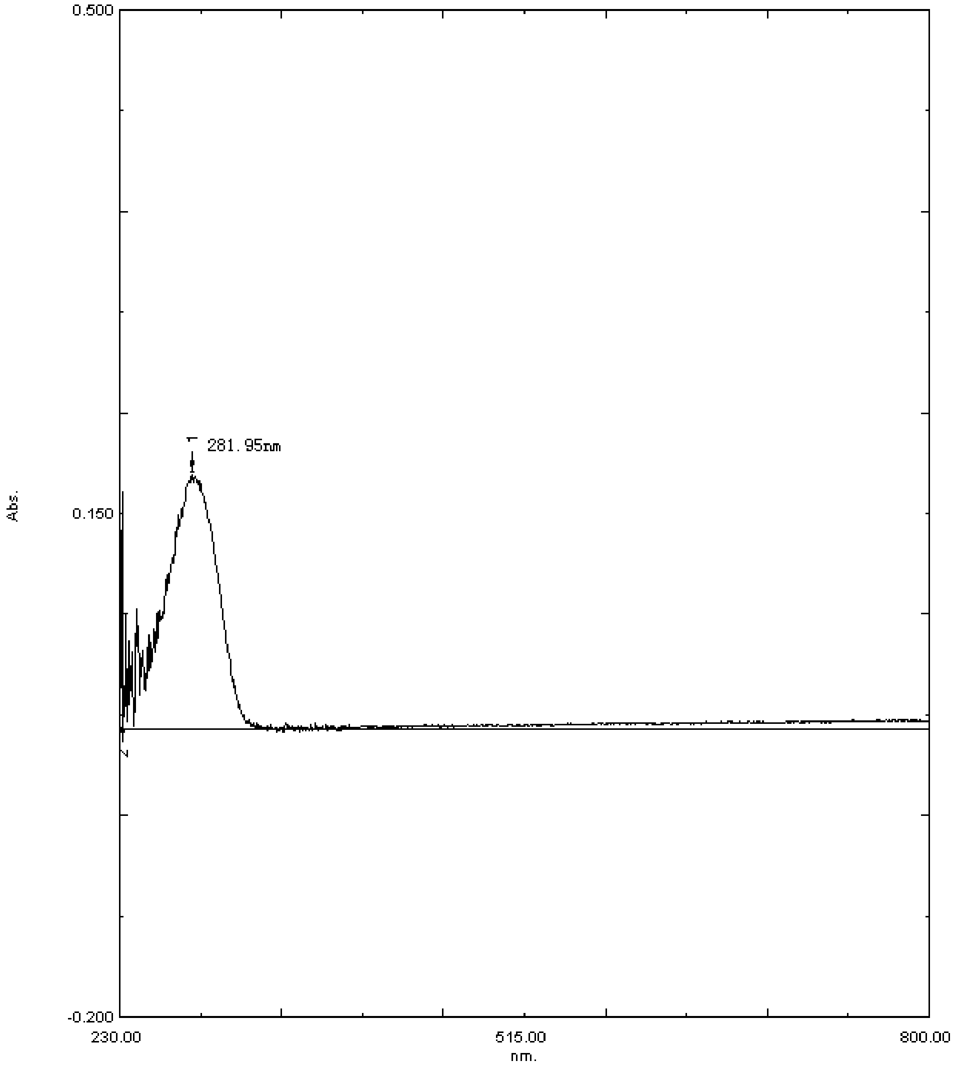

Figure S39. The UV/Vis spectra of compound **4a**

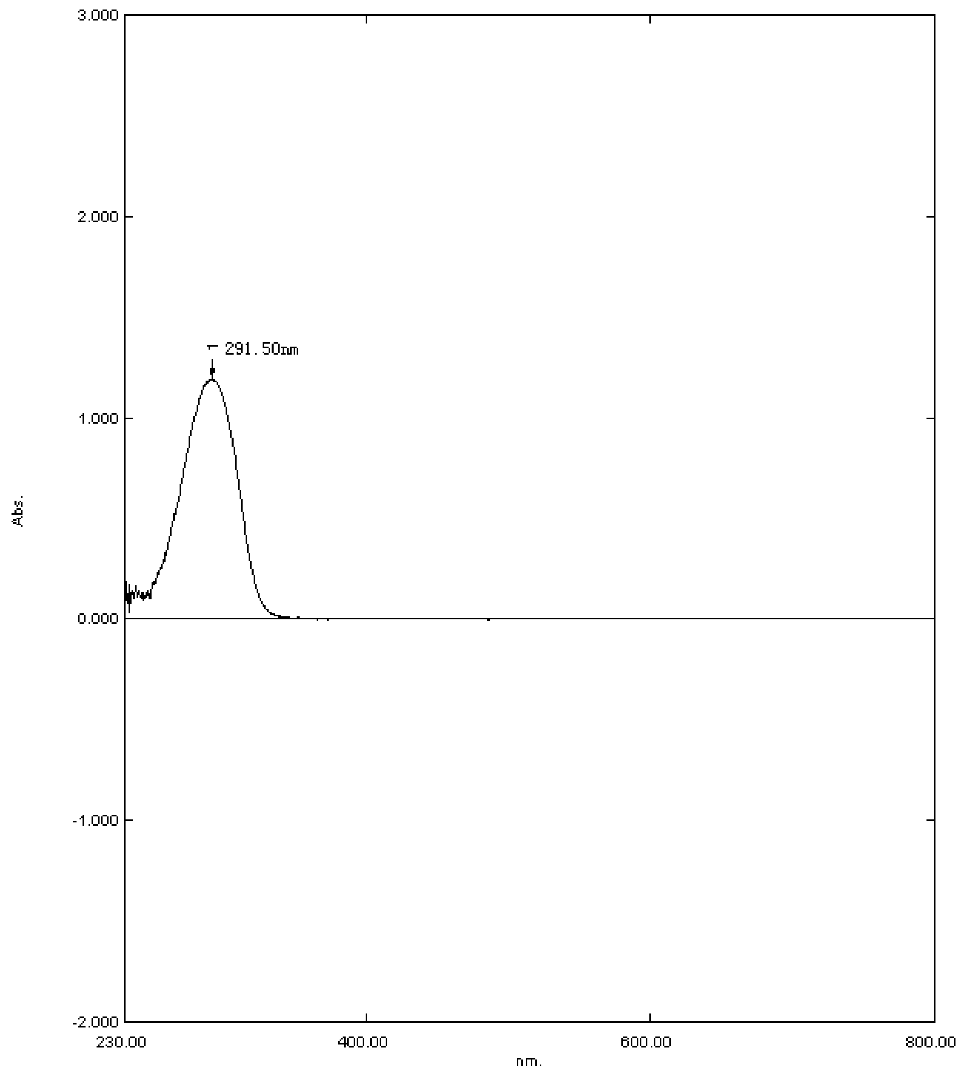

Figure S40. The UV/Vis spectra of compound **4b**
